# Pathways regulating intestinal stem cells and potential therapeutic targets for radiation enteropathy

**DOI:** 10.1186/s43556-024-00211-0

**Published:** 2024-10-10

**Authors:** Si-Min Chen, Bing-Jie Guo, An-Qiang Feng, Xue-Lian Wang, Sai-Long Zhang, Chao-Yu Miao

**Affiliations:** 1 Department of Pharmacology, Second Military Medical University/Naval Medical University, 325 Guo He Road, Shanghai, 200433 China; 2 Yueyang Hospital of Integrated Traditional Chinese and Western Medicine, Shanghai University of Traditional Chinese Medicine, Shanghai, China; 3 Department of Digestive Disease, Xuzhou Central Hospital, Xuzhou, China; 4 School of Medicine, Shanghai University, Shanghai, China

**Keywords:** Radiation enteropathy, Intestinal stem cells, Signaling pathway, Biological targets, Treatment methods

## Abstract

Radiotherapy is a pivotal intervention for cancer patients, significantly impacting their treatment outcomes and survival prospects. Nevertheless, in the course of treating those with abdominal, pelvic, or retroperitoneal malignant tumors, the procedure inadvertently exposes adjacent intestinal tissues to radiation, posing risks of radiation-induced enteropathy upon reaching threshold doses. Stem cells within the intestinal crypts, through their controlled proliferation and differentiation, support the critical functions of the intestinal epithelium, ensuring efficient nutrient absorption while upholding its protective barrier properties. Intestinal stem cells (ISCs) regulation is intricately orchestrated by diverse signaling pathways, among which are the WNT, BMP, NOTCH, EGF, Hippo, Hedgehog and NF-κB, each contributing to the complex control of these cells' behavior. Complementing these pathways are additional regulators such as nutrient metabolic states, and the intestinal microbiota, all of which contribute to the fine-tuning of ISCs behavior in the intestinal crypts. It is the harmonious interplay among these signaling cascades and modulating elements that preserves the homeostasis of intestinal epithelial cells (IECs), thereby ensuring the gut's overall health and function. This review delves into the molecular underpinnings of how stem cells respond in the context of radiation enteropathy, aiming to illuminate potential biological targets for therapeutic intervention. Furthermore, we have compiled a summary of several current treatment methodologies. By unraveling these mechanisms and treatment methods, we aspire to furnish a roadmap for the development of novel therapeutics, advancing our capabilities in mitigating radiation-induced intestinal damage.

## Introduction

Radiation enteropathy, a prevalent complication following radiotherapy for abdominal and pelvic tumors, stems from the direct damage high-energy radiation inflicts upon intestinal tissues, including the small intestine and the colon-rectum [1–3]. This damage affects not only the intestinal mucosa but can penetrate deeper into intestinal structures, instigating a multifaceted pathological process that encompasses acute inflammatory responses and evolves into chronic fibrotic changes [4–6]. Radiation enteropathy can be categorized into acute and chronic phases based on its progression. The acute phase generally appears during or soon after radiotherapy, marked by symptoms such as diarrhea, abdominal pain, and nausea. Conversely, the chronic phase might develop over months to years post-treatment [7, 8]. Currently, there are no standardized and normative treatment protocols for radiation enteropathy, and the primary goal remains symptom alleviation [9, 10]. While these approaches demonstrate some degree of effectiveness, they primarily offer symptomatic relief without addressing the underlying progression of radiation enteropathy. Therefore, further exploration and elucidation of the pathogenesis of radiation enteropathy are essential to provide a theoretical foundation for the discovery of biomarkers, identification of therapeutic targets, and development of novel treatments.

Intestinal stem cells (ISCs), nestled in the specialized microenvironment of intestinal crypts, undertake a vital journey of upward migration and differentiation into the diverse array of intestinal epithelial cells (IECs) that compose the gut lining [11–13]. The crypt's concaved architecture minimizes direct exposure to the harsh digestive milieu, affording them a sanctuary from the abrasive digestive process. Paneth cells, in a dual role of guardian and nurturer, reinforce this protective cocoon. They secrete antimicrobial molecules, which, upon release into the intestinal lumen, collaborate with goblet cell-secreted mucus to constitute a vital component of the mucosal shield that safeguards the small intestinal epithelium [14–16]. Under pathological conditions, Paneth cells exhibit an extraordinary plasticity, capable of reverting to an ISC-like state, a transformation that bolsters the intestinal tissue's regenerative capacity and accelerates repair processes, highlighting their pivotal role in intestinal homeostasis and restitution [17–19]. Throughout the intricate cycle of tissue regeneration, these ISCs are governed by a network of signaling pathways, chief among them being WNT, BMP, NOTCH, EGF, Hippo, Hedgehog and NF-κB [20–23]. The pluripotency of ISCs, empowering them to transform into every type of intestinal cell, renders them indispensable to the restoration and rejuvenation of the intestinal tract following radiation-induced injury [24–26].

In this review, we elaborated on the fundamental architecture of the small intestine, emphasizing the pivotal role of stem cells in injury restoration. Subsequently, we consolidated the prevailing comprehension of the mechanisms underlying radiation enteropathy, complemented with the other factors and potential therapeutic targets. Moreover, we reviewed the current therapeutic strategies devised to address radiation enteropathy. Lastly, we engaged in a discourse addressing the intricate challenges that hinder the advancement of research on the mechanisms of this condition. While there are numerous reports on the regulation of ISCs and crypt homeostasis, most of them consist of independent experiments lacking comprehensiveness. We primarily focus on the molecular mechanisms associated with ISCs in radiation enteropathy and aims to explore potential therapeutic targets and serve as a reference guiding future investigations and innovations in drug development.

## Structure of the small intestine and radiation enteropathy

### Villi and crypts

**Fig. 1:**
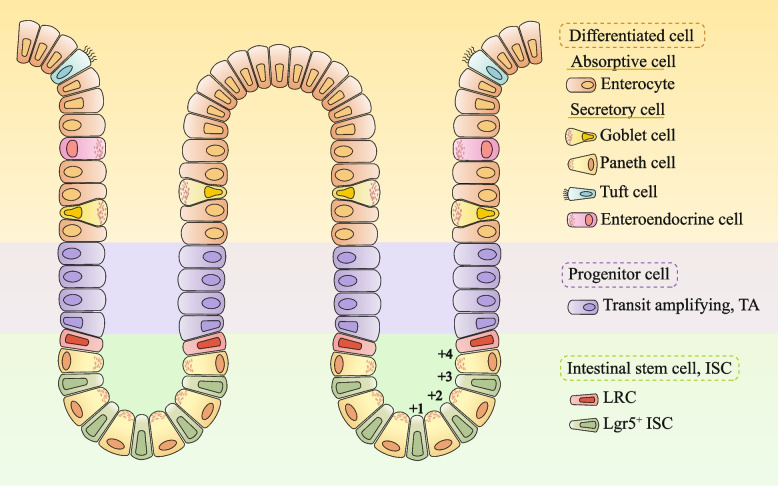
Schematic diagram of small intestinal epithelial tissue structure. The main cell types of intestinal epithelial tissue are stem cells (ISCs), Progenitor cells and Differentiated cells. Label-retaining cells (LRC) and leucine-rich-repeat-containing G-protein-coupled receptor 5 (Lgr5^+^) ISC were intestinal stem cells and transit-amplifying cells (TAC) was progenitor cells. There are two types of differentiated cells: Absorptive cells and Secretory cells. Absorptive cells are Enterocytes; The secretory cells include Goblet cells, Paneth cells, Tuft cells and Enteroendocrine cells

The small intestine's epithelial landscape is featuring myriad crypts interspersed among protruding villi. These crypts, nestled at the base of each villus, shield resident cells from the rigors of the digestive milieu. Within these crypts, intestinal stem cells (ISCs) divide, giving rise to transit-amplifying cells (TACs) that subsequently differentiate into the diverse array of intestinal epithelial cells (IECs) [27–29]. These mature cells can be classified into 6 types: absorptive cells (enterocytes and M cells) and secretory cells (Paneth, goblet, enteroendocrine, and Tuft cells) (Fig. 1). Each IEC type fulfills a unique and indispensable role within the intestinal ecosystem. Consequently, these differentiated IECs are pushed out of the crypts and gradually move towards the tip of the villi. Eventually, these cells undergo apoptosis and are shed into the intestinal lumen, thereby completing their life cycle [30–32]. Research has shown that radiation can destroy the intestinal crypt-villi structure, leading to villi shortening, necrosis and shedding of epithelial cells, and a decrease in the number of crypts [33, 34].

### Intestinal stem cells

Crypt-based columnar cells (CBCs), functioning as the resident ISCs, perpetually divide to fuel the production line of various epithelial cells populating the small intestine [35–37]. Lgr5^+^ CBCs can be labeled by leucine-rich repeat-containing G-protein-coupled receptor 5 (Lgr5), a pivotal player in the WNT signaling cascade. These cells located exclusively at the base of the crypt, surrounded by a cohort of Paneth cells [38] (Fig. 1). Nestled at the distinctive + 4 position within the crypt's proliferation zone, resides a specific type of cell. They are designated as label-retaining cells (LRCs) [39, 40]. LRCs play a pivotal role in the healing of intestinal, catalyzing epithelial regeneration post-injury [41–43]. However, the identification of a specific marker for LRCs is still a matter of debate. Research has hinted at candidates such as Bmi1, Tert, Hopx, and Lrig1 as tags for LRCs instrumental in IECs restoration post-damage [41–44]. Yet, a twist in the tale emerged with the revelation that even Lgr5^+^ ISCs can express these same markers, complicating efforts to isolate LRCs based solely on these markers [45]. Lgr5^+^ ISCs display a notable susceptibility to irradiation, leading to a substantial reduction in their population. Conversely, the number of LRCs situated at the crypt + 4 position is observed to increase post-irradiation [46]. These LRCs characteristically express KLF4, a zinc finger transcription factor (Krüppel-like factor 4), which governs the cell cycle by decelerating cellular proliferation. Research has highlighted that conditional ablation of KLF4 in murine models impedes the regenerative capacity of the intestinal epithelium post-irradiation, implicating KLF4 as a pivotal factor in preserving the quiescent state of LRCs and their inherent radioresistance [47] (Table 1). While it is widely accepted that Lgr5^+^ ISCs are sensitive to irradiation, some studies have also indicated that they possess a certain degree of radiation resistance. Not all Lgr5^+^ ISCs undergo apoptosis following irradiation, and the remaining cells serve as an important source for intestinal epithelial regeneration [48]. Furthermore, the quiescent or slowly dividing LRCs can assume the ISC role, thereby serving as a cellular backup for the comprehensive renewal of the intestinal lining [49, 50].

**Table 1:** Potential intervention targets after radiation

Signaling pathway	Intervention factor	Original function	Effects after irradiation	Ref
WNT	R-spondin1	Agonist of canonical WNT	Survival rate↑; Crypt cells↑; IECs↑; Lgr5^+^ ISCs↑	[51]
	LIG4	DNA double-strand break repair factor	WNT signal↑; IECs radioresistance↑	[52]
	URI	Genome integrity regulator	Intestinal damage↓; LRCs radioresistance; WNT signal ↓	[53]
	BCN057	Antineoplastic small molecular agent	Intestinal damage↓; WNT signal↑; Lgr5^+^ ISCs↑; IECs↑	[54]
	Rebamipide	Gastric mucosal protective agent	Intestinal damage↓;Intestinal barrier↑; MMP-9↓; IECs↑	[55]
	Podophyllotoxin & rutin	Endogenous cellular antioxidant regulator	Crypt cells↑; c-Myc↑; Survival rate↑; DNA damage↓	[56]
BMP	Bmpr1a	Maintain ISC stemness during homeostasis	Lgr5^+^ ISCs↑	[57]
	ANGPTL2	promote angiogenesis and development	Lgr5^+^ ISCs↑	[58]
	MMP-17	Maintain ISC stemness during homeostasis	SMAD4↑; BMP signaling↑; MMP17↓, Intestinal damage↓	[59]
NOTCH	Ghrelin	Appetite stimulating polypeptide	Dll 1, 3, 4↑; Villus length↑; Crypt cells↑; Intestinal barrier↑	[60]
EGF	IL-33	immune responses and inflammatory reactions	IECs↑; Lgr5^+^ ISCs↑	[61]
	IL-22	immune responses and inflammatory reactions	crypt numbers↑; Lgr5^+^ ISCs↑	[62]
	HB-EGF	Ligands for EGFR and ErbB4	Crypt cells↑;Intestinal damage↓; Intestinal barrier↑	[63]
	CA	TCM with extensive medicinal value	Intestinal barrier↑	[64]
Other Factors	KGF	Epithelium specific growth factor	Lgr5^+^ ISCs↑; Crypt cells↑	[65]
	GLP-2	Nutrient absorption polypeptide	Survival time↑; Inflammation ↓;Crypt cells↑; IECs apoptosis↓	[66]
	5-HT	Serotonin	Intestinal regeneration↑	[67]
	VA	Short chain fatty acid	Weight↑; Inflammation↓;	[68, 69]
	HA-β-CD	Supramolecular delivery platform	Intestinal barrier↑; Microbiome balance	[70]
	ADSC	Adipose-derived stem cell	fibrosis↓; Microbiome balance	[71]
	KLF4	Cellular process regulator	Crypt cells↑; Survival rate↑; LRCs↑	[47]

*URI* unconventional prefoldin RPB5 interactor, *CA* centella asiatica, *TCM* traditional chinese medicine, *KGF* keratinocyte growth factor, *GLP-2* glucagon-like peptide 2 analogue, *5-HT* 5-hydroxytryptamine, *VA* valeric acid, *KLF4* Krüppel-like factor 4, *HA-β-CD* hyaluronic acid (HA) and β-cyclodextrin (HA-β-CD), *ADSC* adipose-derived mesenchymal stem cells, *IECs* intestinal epithelial cells, *ISCs* intestinal stem cells, ↑ increase, ↓ decrease

## Signaling pathways of ISCs under radiation conditions and potential therapeutic targets

### WNT signaling pathway

#### WNT regulation under physiological condition

**Fig. 2:**
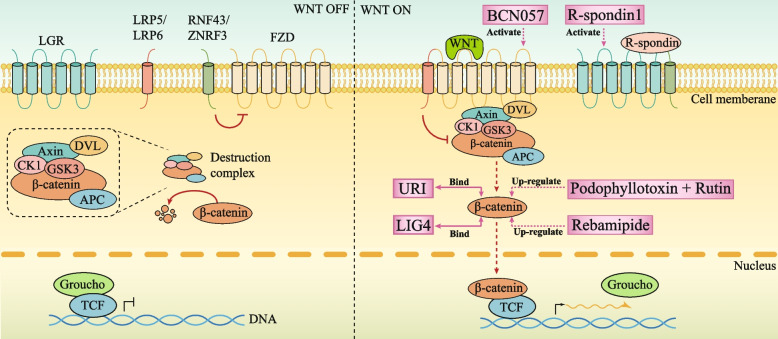
WNT signalling pathways in the intestinal crypt and potential therapeutic targets. Sustained activation of the WNT signaling pathway is dependent on the presence of R-spondin. R-spondin binds to LGR family receptors and also binds to RNF43-ZNRF3, which in turn stabilizes FZD expression. Upon binding to FZD and LRP5-LRP6, the WNT pathway is activated. This activation leads to the activation of the intracellular scrambling protein Dvl (Dishevelled), a crucial component in the WNT signaling cascade. Dvl inhibits the phosphorylation of β-Catenin by disrupting the complex composed of Axin, adenomatous polyposis coli (APC), casein kinase 1 (CK1), and glycogen synthase 3 (GSK3). Accumulated β-catenin then enters the nucleus and promotes transcription by binding to T-cell factor (TCF) and displacing Groucho. The potential targets and their mechanisms of action have been marked with pink boxes

WNT signaling activity reaches its apex in ISCs situated at the crypt's base, tapering off in a distinct gradient as one moves from the crypt towards the villus [72, 73] (Fig. 2). In the absence of R-spondin, RNF43 or ZNRF3 can initiate the ubiquitination and subsequent dismantling of the frizzled (Fzd) receptor upon its membrane binding [74–76]. Under conditions where no WNT ligand is present (WNT Off state), β-Catenin adheres to a cytoplasmic complex comprised of Axin, adenomatous polyposis coli (APC), casein kinase 1 (CK1), and glycogen synthase 3 (GSK3), leading to its phosphorylation and eventual degradation. Conversely, upon the engagement of the WNT ligand with its membrane receptors Fzds and auxiliary receptors LRP5 and LRP6 (Wnt On state), the intracellular mediator Dvl (Dishevelled – a pivotal player in the WNT signaling cascade) becomes activated, effectively halting the complex's phosphorylation of β-Catenin. Accumulated β-Catenin then migrates to the nucleus, forming complexes with LEF/TCF transcription factors. This interaction triggers the transcription of a myriad of downstream targets, including but not limited to c-myc, Cyclin D1, and Lgr5 [77–79] (Fig. 2).

Suppression of the WNT signaling pathway via diverse mechanisms – including inhibition of the WNT ligand [80], downregulation of β-Catenin [81], silencing of the proto-oncogene c-myc [82], or knockdown of the WNT signaling nuclear transcription factor Tcf4 [83]—induces the demise of Lgr5-positive ISCs and the consequent erosion of small intestinal crypts. This chain of events obstructs the genesis of normal intestinal epithelial cells (IECs), thereby undermining intestinal integrity. Conversely, amplification of WNT signaling, achieved by methods such as administering R-spondin protein (a WNT pathway activator) to mice [84], concurrent deletion of RNF43 and ZNRF3 (negative regulators of WNT signaling) [75], systemic elimination of mouse APC (Adenomatous Polyposis Coli) [85] or targeted APC deletion in Lgr5^+^ ISCs [86], provokes excessive proliferation of Lgr5^+^ ISCs within the small intestine, marked crypt hyperplasia, attenuated differentiation of IECs, and fosters tumorigenesis. Collectively, these observations underscore the fundamental role of WNT signaling in orchestrating ISC proliferation, differentiation, and tissue homeostasis, emphasizing that dysregulation of this pathway is instrumental in tissue malfunction and the onset of intestinal tumors.

#### WNT regulation after irradiation and potential therapeutic targets

Following irradiation, the impairment of the crypt-villus architecture in the intestinal epithelium and the consequent loss of crypt cells undermine the effective renewal and replacement of the intestinal lining, giving rise to radiation-induced enteropathy. Some reports indicate that the WNT pathway is upregulated following radiation exposure as a mechanism to counteract the damage inflicted on the small intestine [51, 52]. After irradiation, mice exhibit upregulated expression of R-spondin1 in serum, indicating the activation of the WNT. Furthermore, exogenous administration of R-spondin1 has been shown to reduce radiation-induced apoptosis and elevate the survival rates (Table 1, Fig. 2) [51]. This study suggests that R-spondin1 may serve as a therapeutic target for radiation enteropathy. However, this work focuses solely on the phenotype and does not explore in depth how the WNT pathway regulates small intestinal crypt cells to confer radiation resistance. Another study explored the regulatory mechanisms of the WNT and discovered that LIG4, a key ligase in the repair of DNA double-strand breaks, is a direct target of β-catenin. During radiation-induced intestinal regeneration, the WNT signaling pathway is activated, with LIG4 primarily expressed in the crypts and upregulated in stem cells (ISCs). Further blocking of LIG4 increases the sensitivity of the small intestine to radiation (Table 1, Fig. 2) [52]. This work suggests that DNA damage repair is one of the mechanisms by which the WNT pathway provides radiation protection, and intervening with LIG4 may offer better radiation protection compared to targeting other components of the WNT pathway.

Besides the aforementioned mechanisms, does WNT pathway have any other protective mechanisms? Another study provides us with new insights. Intriguingly, its findings diverge from earlier research, revealing that suppressing WNT subsequent to radiation exposure exerts a protective influence on intestinal tissues [53]. The study reports that URI (Unconventional prefoldin RPB5 interactor, a crucial molecular chaperone) effectively mitigates the severity of radiation-induced damage in the small intestine. Its specific mechanism entails URI directly binding to β-catenin within LRC, which subsequently impedes the nuclear translocation of β-catenin. This process downregulates the WNT pathway, ultimately conferring protection to the intestinal tissues (Table 1, Fig. 2). This study underscores that inhibiting the WNT pathway can confer protective benefits to the intestines post-radiation exposure. Notably, while its conclusions diverge from previous research on WNT pathway alterations, they highlight the ambiguity surrounding the role of even a well-established pathway like WNT in radiation-induced enteropathy. This finding underscores the imperative for further research to unravel the intricate mechanisms underlying radiation-induced enteropathy, thereby advancing therapeutic strategies for this condition.

Beyond the above pathway studies, there are also small molecule compounds and drugs, originally used for treating other conditions, that are being investigated for their potential to treat radiation-induced enteropathy. Recent reports have highlighted the capacity of the small-molecule anti-tumor compound BCN057 to upregulate the WNT signaling pathway within intestinal crypts, significantly enhancing stem cell proliferation and regenerative potential after radiation [54]. This activation serves to alleviate the devastation inflicted by acute radiation enteropathy, all while abstaining from augmenting the radioresistance of tumor cells. BCN057 emerges as a promising mitigatory agent for managing acute radiation-induced enteropathy, poised to enhance the efficacy of abdominal radiotherapy regimens and mitigate intestinal adverse effects (Table 1, Fig. 2). Another study shows that Rebamipide, a medication commonly prescribed for the treatment of gastric ulcers and gastritis, has demonstrated additional therapeutic potential in models of radiation enteropathy [55]. It has been discovered to enhance the expression of β-Catenin, thereby fostering intestinal proliferation and regeneration. Moreover, Rebamipide exerts an anti-inflammatory influence by suppressing the production of matrix metalloproteinase-9 (MMP-9), a prominent pro-inflammatory mediator. This dual mechanism not only mitigates the inflammatory reaction but also facilitates the restoration of the intestinal barrier integrity compromised by radiation exposure (Table 1, Fig. 2).

Additionally, certain natural extracts are being investigated for their potential in treating radiation-induced enteropathy. For instance, a synergistic blend of podophyllotoxin and rutin has been shown to not only modulate the endogenous cellular antioxidant defense system but also to be effective in treating radiation-induced enteropathy [56]. Research indicates that this blend facilitates the nuclear translocation of β-catenin, triggering the activation of downstream proteins like c-Myc, Survivin, and Nanog, thereby stimulating the proliferation of Lgr5 cells. Moreover, the combination of these two compounds can also reduce radiation-induced DNA damage in the small intestinal crypts. In comparison to the currently approved anti-radiation agent, Amifostine [87], this combination has shown a superior antitumor response (Table 1, Fig. 2). While these three agents—BCN057, Rebamipide, and the podophyllotoxin-rutin blend—have each shown encouraging outcomes in preclinical models, it is critical to recognize that a formidable journey remains before they can transition into clinically viable pharmaceuticals.

### BMP signaling pathway

#### BMP regulation under physiological condition

**Fig. 3:**
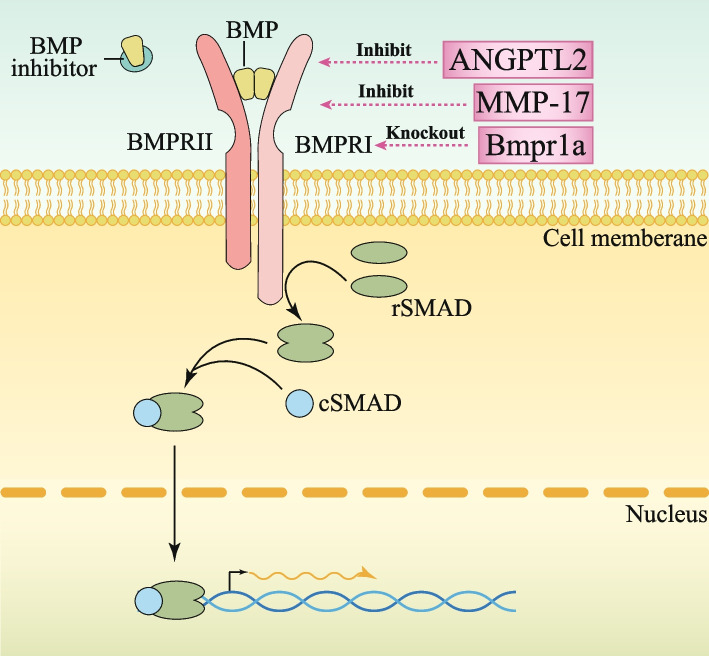
BMP signalling pathways in the intestinal crypt and potential therapeutic targets. BMP signaling pathway induces the dimerization of BMP type I and type II receptors (BMPR1 and BMPRII), resulting in the phosphorylation and subsequent dimerization of receptor-regulated mothers against decapentaplegic homologues (rSMADs). These rSMADs then form heterotrimeric complexes with common SMADs (cSMAD or SMAD4) and translocate to the nucleus. Within the nucleus, this complex regulates the expression of target genes. The potential targets and their mechanisms of action have been marked with pink boxes

BMP (bone morphogenetic protein) is a member of the TGF-β (Transforming Growth Factor β) superfamily [88–90]. The conventional BMP signaling cascade engages a dual-receptor system comprising Serine-Threonine Kinase Receptors of type I (BMPR I) and type II (BMPR II) [91, 92]. Upon binding of BMP ligands to BMPR II, it sets in motion a cascade leading to the phosphorylation of BMPR I. This activated BMPR I further phosphorylates a trio of SMAD proteins—SMAD1, SMAD5, and SMAD8. These phosphorylated SMADs, in conjunction with SMAD4, constitute a transcriptional regulatory complex that migrates into the nucleus, orchestrating the expression of a myriad of downstream target genes, thereby underscoring the intricacy and significance of BMP signaling in cellular regulation and differentiation [93, 94] (Fig. 3). BMP signaling cascade has been implicated in restraining intestinal tumorigenesis. Studies have documented that in Apc-deficient mice, the inactivation of BMP signaling follows the knockout of SMAD4, exacerbating the progression of small intestinal and colonic tumors [95, 96]. Of additional note, the loss of SMAD4 has been demonstrated to subvert the protective, tumor-suppressive function of BMP signaling, converting it into a tumor-promoting entity, underscoring the delicate balance and functional duality of BMP signaling in intestinal homeostasis and disease [97, 98].

In contrast to the graded distribution of WNT signaling intensity, mesenchymal cells nestled in the crypt's lower stratum secrete a panoply of BMP inhibitors, including Noggin, Gremlin 1, Gremlin 2, and Chordin-like 1. Consequently, BMP signal strength exhibits an ascending gradient from the crypt towards the villi [99, 100] (Fig. 3). Research has attested that the surplus expression of Noggin inhibits the BMP signaling cascade, culminating in the aberrant emergence of numerous ectopic crypts spanning both crypt and villus regions in mice [101]. Parallel findings were found by a separate study wherein the overexpression of Gremlin 1 within the mouse intestinal epithelium yielded analogous results [102]. These revelations collectively intimate the inhibitory capacity of BMP signaling in suppressing cryptogenesis and the development of polyps. Beyond its role in curbing ISCs proliferation by tempering WNT signaling, BMP signaling has also been shown to exert direct repression on the hallmark genes of ISCs, intimating a distinct modality by which BMP signaling interferes with the self-renewal of Lgr5^+^ ISCs, operating independently of its regulatory influence on the WNT pathway [103, 104].

#### BMP regulation after irradiation and potential therapeutic targets

Given that under normal physiological conditions, the activity of the BMP pathway is inherently opposed to that of the WNT pathway and is weak within the crypts, the BMP signal becomes even more undetectable after radiation. However, there are still reports showing that inhibiting the BMP pathway after radiation can foster the repair and regeneration of the small intestinal crypt. Specifically, by targeting and knocking out Bmpr1a to suppress the BMP pathway subsequent to radiation, a notable enhancement in the proliferation of Lgr5^+^ stem cells has been observed, underscoring its potential therapeutic value [57]. Moreover, the research delved further into the intricate mechanisms, revealing that BMP employs downstream proteins Smad1/Smad4 to recruit histone deacetylase HDAC1 to the promoters, effectively suppressing stem cell proliferation. Conversely, the ablation of Smad4 was observed to stimulate stem cell regeneration (Table 1, Fig. 3). Complementary to this, another study has illuminated the protective function of angiopoietin-like protein 2 (ANGPTL2) in intestinal radiation injury, a mechanism mediated through regulation of the BMP signaling pathway [58]. The research divulged that ANGPTL2 is synthesized in the intestinal subepithelial myofibroblasts, where it operates in an autocrine manner. Via the integrin α5β1/NF-κB signaling cascade, ANGPTL2 suppresses BMP expression, which in turn, nurtures the multiplication of Lgr5-positive stem cells and accelerates intestinal recovery post-radiation insult (Table 1, Fig. 3). An additional study has demonstrated that inhibition of the BMP pathway fosters stem cell proliferation, whereas enhancing BMP levels attenuates the repair mechanisms following radiation-induced damage. Matrix Metalloproteinase-17 (MMP-17), an inhibitor of the BMP signaling pathway expressed by smooth muscle cells residing at the crypt base, intriguingly, facilitates stem cell proliferation [59]. In the context of radiation-induced intestinal epithelial injury, the absence of MMP-17 was found to hinder the completion of the repair process (Table 1, Fig. 3). Collectively, these findings underscore the intricate dance between BMP signaling and stem cell dynamics in the context of radiation response and tissue regeneration.

### NOTCH signaling pathway

#### NOTCH regulation under physiological condition

**Fig. 4:**
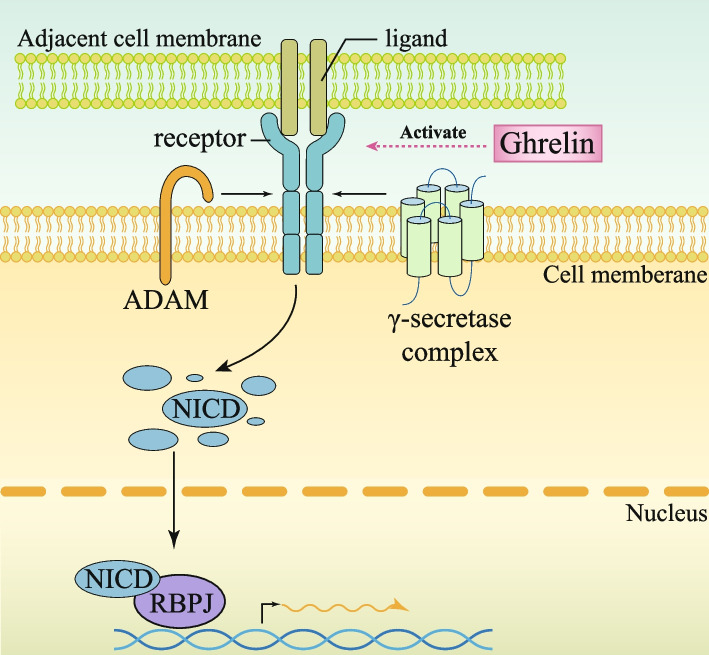
NOTCH signalling pathways in the intestinal crypt and potential therapeutic targets. NOTCH signaling is initiated when NOTCH receptors bind to NOTCH ligands. The synthesis of NOTCH receptors takes place in the endoplasmic reticulum, followed by processing in the Golgi apparatus. Subsequently, the receptors are transported to the cell surface where they can bind and interact with NOTCH ligands present on adjacent cells. The transmembrane region of the receptor undergoes cleavage by ADAM (a disintegrin and metalloprotease), and then further cleavage occurs through γ-secretase protease. Once the receptor is cleaved, the NOTCH intracellular domain (NICD) is released and translocated to the nucleus where it binds to recombination signal binding protein for immunoglobulin kappa j region (RBPJ), thereby activating downstream genes. The potential targets and their mechanisms of action have been marked with pink boxes

The NOTCH signaling cascade exerts its multifaceted influence through the interaction of transmembrane ligands and receptors between adjacent cells. Within the microcosm of the small intestine, 4 NOTCH receptors (NOTCH1-4) and 5 ligands (DLL1, 3, 4 and JAGGED1, 2) hold exclusive residency in the crypt domain. The activity of this NOTCH signaling cascade is spatially confined to the crypt region, as documented by studies [105–107] (Fig. 4). Ligand binding prompts a sequential cleavage of the receptor: first by ADAM metalloproteases (S2 cleavage), followed by γ-secretase-mediated processing (S3 cleavage). Post-cleavage, the NOTCH intracellular domain (NICD) infiltrates the cellular nucleus [108, 109]. Here, NICD aligns with CSL (CBF1/Rbpj) and the transcriptional coactivator MAML, forming a potent trimeric transcriptional activator complex. This ensemble acts as a molecular switch, flipping on the transcription of downstream NOTCH targets, notably including Hes and Atoh1 (Math1) [110–112] (Fig. 4). This intricate interplay orchestrates a symphony of cellular responses, underscoring the pivotal role of NOTCH signaling in intestinal homeostasis and beyond.

The NOTCH signaling pathway has emerged as a linchpin in preserving the proliferative capacity of intestinal crypts. Research has illuminated that heightened expression of Notch 1 and Notch 2 in murine models fosters augmented NOTCH signaling, thereby accelerating the division of intestinal stem cells (ISCs) and absorptive precursor cells. Strikingly, this signaling surge is conspicuously absent in secretory precursor cells [113, 114]. An investigation of small intestinal crypts disclosed a compelling transformation: in the absence of either Notch 1 or Notch 2, proliferating cells were destined to mature into secretory lineage goblet cells [115, 116]. Furthermore, by directly suppressing the transcription of Atoh1, Hes1 curtails the differentiation of secretory cells and restrains ligand synthesis. This intricate regulatory mechanism is pivotal for maintaining equilibrium between secretory and absorptive cell populations, while concurrently sustaining a stable reservoir of ISCs at the crypt base [117, 118]. Interference with NOTCH signaling via administration of a γ-secretase inhibitor disrupts the delicate homeostasis of IECs, leading to the depletion of ISCs, a decline in cellular proliferation, and an increase in apoptosis. Concomitantly, precocious differentiation of epithelial progenitor cells into Paneth and goblet cells is noted [119, 120], echoing findings from prior investigations in ADAM metalloproteinase-deficient murine models [40]. Conversely, excessive activation of NOTCH signaling within IECs gives rise to a marked proliferation of cells; however, this hyperstimulation concurrently triggers the loss of differentiated secretory cell populations, highlighting the delicate balance required for proper ISCs function and intestinal epithelium maintenance [121, 122]. Additionally, Rfng and Lfng have been identified to amplify NOTCH signaling within ISCs by augmenting the availability of cell surface ligands, specifically Dll1 and Dll4. Both these glycosyltransferases exert an inhibitory effect on the differentiation trajectory of transit-amplifying cells (TACs) towards endocrine, Tufted, and goblet cell fates [123, 124]. Collectively, these revelations paint a picture where Notch act in concert to shepherd ISCs proliferation, dictate cell fate decisions towards distinct lineages, and facilitate the restorative response following injury.

#### NOTCH regulation after irradiation and potential therapeutic targets

In light of the pivotal function of the NOTCH signaling pathway within the intestinal tissue, a growing body of research has begun to focus on its alterations in radiation-induced intestinal disease. Several studies have indicated a notable upregulation of the NOTCH signaling pathway following radiation exposure [125–128]. Following radiation exposure, organoids positive for proliferation markers like Lgr5 or Ki67 exhibit a resurgence of Notch activity, reviving a pathway previously dormant in secretory progenitors or Paneth cells [125]. Concurrently, radiation stimulates a cascade of Notch target gene activations, boosting the expression of proliferative transcription factors in Paneth cells devoid of typical stemness traits, thereby conferring upon them pluripotency and dedifferentiation potential. Of note, the detection of YAP and Stat3 specifically in irradiated Paneth cells, in comparison to non-irradiated counterparts, further accentuates the narrative [125]. Another report utilizing organoids as research models has also revealed that during organoid formation, radiation-induced NOTCH activation predominantly stems from an elevation in NOTCH receptor expression, diverging from the conventional activation of the Wnt/β-catenin pathway in Paneth cells. This unique mechanism propels these differentiated cells into a state of dedifferentiation, metamorphosing them into stem cell-like precursors instrumental in revitalizing and repopulating the villus epithelium [126]. Conversely, suppression of NOTCH signaling compromises the survival of crypt stem cells following radiation insult, emphasizing the fundamental role of NOTCH signaling as a governing axis in ISC proliferation, differentiation, tissue repair, and the overall sustenance of intestinal epithelial cell homeostasis [127].Furthermore, it has been documented that in mouse intestines deficient in Notch1 and Notch2, the regenerative capacity of intestinal crypts post-radiation is significantly hampered, implying an elevated necessity for NOTCH signaling in the aftermath of injury [128]. These evidences collectively intimate that the rewiring of the NOTCH pathway post-radiation damage may constitute a vital component of a radiation-defense signaling network, intertwining cellular survival, proliferation, DNA restoration, and epithelial rejuvenation.

Beyond the aforementioned research on the NOTCH pathway, there is another report suggesting that protein with other functions in the intestine can exert a radioprotective effect by upregulating the NOTCH pathway. It has been found that Ghrelin, an endogenous peptide hormone synthesized by ghrelinergic cells in the gastrointestinal tract, induces the proliferation of irradiated IECs through activation of Notch [60]. This hormone exhibits promise in mitigating intestinal damage inflicted by acute radiation enteropathy in murine models and in restoring the intestinal barrier integrity compromised by radiation exposure (Table 1, Fig. 4). Moreover, exogenous administration of Ghrelin also reduced the severity of radiation-induced intestinal injury in a mouse model, thereby positioning Ghrelin as a prospective therapeutic intervention for managing acute radiation-induced intestinal injuries. However, it is essential to underscore that the exploration into the mechanism of action of Ghrelin remains insufficiently profound. Consequently, additional efforts are warranted to definitively establish the functions and intricate mechanisms of this protein.

### EGF signaling pathway

#### EGF regulation under physiological condition

**Fig. 5:**
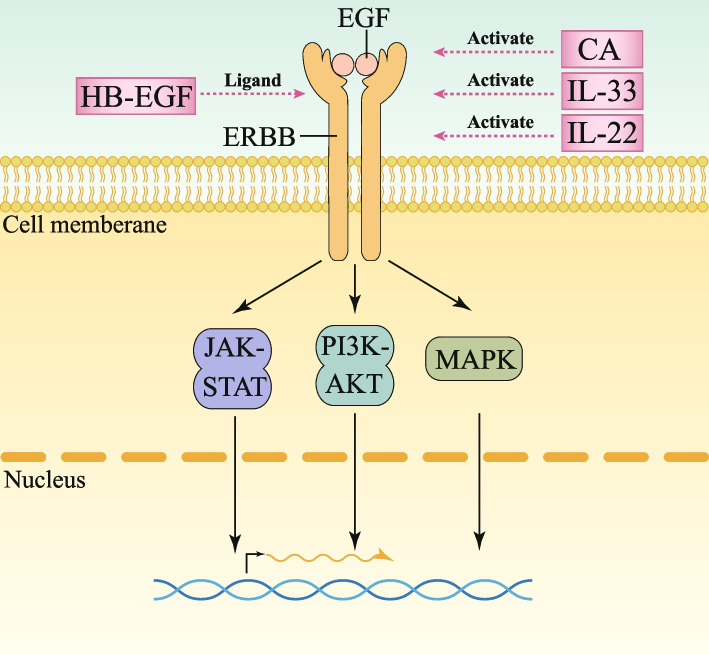
EGF signalling pathways in the intestinal crypt and potential therapeutic targets. EGF: EGF stimulates the EGF receptor (ERBB), initiating the mitogen-activated protein kinase (MAPK) cascade. This cascade leads to the phosphorylation of various nuclear targets by ERK, which promotes cell proliferation and inhibits apoptosis. Simultaneously, EGF signaling enhances the interaction between phosphocarnosine 3-kinase (PI3K) and converts PIP2 to PIP3. Furthermore, the activated EGF receptor complex recruits Janus kinases (JAKs). The potential targets and their mechanisms of action have been marked with pink boxes

The EGF Ligand family is comprised of eleven structurally akin proteins: EGF itself, Transforming Growth Factor Alpha (TGF-α), Amphiregulin (AREG), Epigen (EPGN), Heparin-Binding EGF-Like Growth Factor (HB-EGF), Epiregulin (EREG), Betacellulin (BTC), and the quartet of Neuregulins (NRG1, 2, 3, 4). Mesenchymal cells within the intestine secrete NRG1, NRG2, AREG, and EREG, which exert their effects on epithelial cells through a paracrine mode of action. Conversely, IECs produce EGF, BTC, HB-EGF, and TGF-α, regulating their own functions in an autocrine fashion [129–131]. Embedded within the tyrosine kinase receptor family are the EGF receptors, namely EGFR (alias ErbB1), ErbB2, ErbB3, and ErbB4. EGFR finds expression in ISCs and TACs, while ERBB2 and ERBB3 are omnipresent across the crypt-to-villus architecture of the small intestine [132–134]. This ensemble of eleven ligands can be classified into four groups based on their varied receptor binding preferences. These ligands proceed to engage with distinct receptors – excluding ErbB2, which awaits a confirmed ligand partner – forming receptor dimers that efficaciously initiate intracellular signaling cascades, including Mitogen-Activated Protein Kinase (MAPK), Phosphoinositide 3-Kinase-AKT (PI3K-AKT), and Janus Kinase-Signal Transducer and Activator of Transcription (JAK-STAT) pathways, thereby orchestrating a symphony of cellular responses vital to intestinal homeostasis and function [135, 136] (Fig. 5).

The EGF signaling pathway is instrumental in regulating diverse intestinal functions. Specifically, by activating EGFR, it propels intestinal cell proliferation, expediting the repair and restoration of intestinal damage [137]. Moreover, this pathway reinforces intestinal barrier function, acting as a shield against the penetration of harmful substances, thereby safeguarding gut health [138]. The maintenance of an intact intestinal barrier is crucial, and the EGF signaling pathway reinforces this defense mechanism by fostering tight junctions between intestinal cells and enhancing the formation of mucus layers. Beyond its role in cellular proliferation and barrier function, the EGF signaling pathway also modulates intestinal inflammation [139]. By suppressing the generation and release of inflammatory factors, it mitigates inflammatory responses within the gut, fostering its recovery and healing processes.

#### EGF regulation after irradiation and potential therapeutic targets

Given the significant role of the EGF signaling pathway in promoting intestinal cell proliferation, repair, and protection against damage, an increasing number of studies are focusing on its alterations in radiation-induced intestinal diseases, with the aim of identifying potential therapeutic targets [61, 62, 140, 141]. Upon reviewing current literature, the EGF pathway is generally upregulated in radiation enteropathy. It has been documented that IL-33 plays a pivotal role in facilitating the regeneration of intestinal epithelium and enhancing the recovery of the intestinal crypt stem cell niche subsequent to radiation-induced damage [61] (Table 1, Fig. 5). Specifically, in response to radiation exposure, intestinal stem cells (ISCs) produce IL-33, and their strategic positioning in close proximity to Paneth cells enables efficient transmission of IL-33 signals to adjacent Paneth cells. As a result, IL-33 exerts a stimulatory effect, accelerating the proliferation of intestinal epithelial cells, maintaining crypt integrity, and promoting the repair of ISCs post-radiation through the upregulation of EGF expression in Paneth cells. A separate study utilizing organoid models also revealed a similar trend. Research has convincingly demonstrated that IL-22 significantly enhances the survival rate of crypt cells subsequent to radiation exposure [62] (Table 1, Fig. 5). Notably, IL-22 further augments the quantity of organoids generated from individual cells post-radiation, with this protective effect becoming increasingly evident as radiation doses escalate. Remarkably, this protective mechanism remains resilient, enduring even seven days post-radiation. Conversely, dampening the EGF signaling pathway inversely correlates with a decline in intestinal crypt numbers, impedes ISC proliferation, and diminishes the regenerative capability of intestinal tissue [135]. Nonetheless, unchecked activation of the EGFR holds the ominous potential to fuel tumor development, underscoring the necessity for a delicate balance in its regulation [142].

In addition to the aforementioned physiological regulations, reports have demonstrated that direct exogenous intervention yields significant protective outcomes in models of radiation enteropathy. HB-EGF (Heparin-binding epidermal growth factor-like growth factor), a glycoprotein member of the EGF family, exhibits the capability to stimulate crypt proliferation and mitigate intestinal damage subsequent to radiation exposure (Table 1, Fig. 5) [63]. While the current study may be considered somewhat superficial in its scope, lacking a profound exploration of the underlying mechanisms, it nonetheless presents an innovative perspective that offers a promising new avenue for the treatment of radiation enteropathy. In comparison, a separate study has undertaken a more thorough examination of the therapeutic mechanisms, offering a deeper understanding of the process. Centella asiatica (CA), a medicinal herb, has proven efficacious in remedying radiation-induced disruptions through modulating EGF activity, reinforcing its value in this context [64]. Additionally, the research unveiled the beneficial therapeutic impacts of CA-mediated endothelial paracrine signaling in a murine model of radiation-induced enteritis, notably coinciding with the restoration of the epithelial barrier, underscoring its potential as a therapeutic target (Table 1, Fig. 5).

### Others

#### Hippo signaling pathway

Apart from the aforementioned four prevalent pathways contributing to radiation enteropathy, there exist two additional pathways (Hippo and Hedgehog signaling pathway) that are instrumental, particularly in the intricate regulation of small intestinal stem cells. The Hippo signaling cascade holds a paramount position in preserving the normal gut homeostasis of the intestinal tract. Conditional abrogation of Hippo signaling culminates in tissue hyperplasia and tumorigenic predisposition [143–145]. This Hippo pathway intricately intersects with several pivotal signaling networks within the intestinal crypt, notably including the WNT pathway, to ensure the principle of signaling intensity that is conducive to both tissue renewal and tumorigenesis [146–148]. The Hippo pathway exerts a braking effect on WNT signaling by repressing a cadre of canonical WNT target genes alongside cellular identifiers of ISCs, such as LGR5 and Axin2 [149]. Additionally, activation of Hippo signaling leads to the transcriptional induction of antagonists of WNT, such as Dkk 1, Bmp 4, and Wnt 5α [150]. Investigations have also illuminated the Hippo pathway's regulatory influence on elevating Hes1 gene expression, thereby sculpting the NOTCH signaling relay [151]. Moreover, in the context of intestinal regeneration, Hippo signaling partakes in the bolstering of EGF signaling by modulating the expression of EGFR ligands, underscoring its multifaceted role in integrating and harmonizing critical pathways governing gut homeostasis and repair [152]. Based on the above research findings, it becomes evident that the Hippo pathway primarily exerts its regulatory influence on radiation enteropathy by engaging in crosstalk with the four previously established classical pathways.

#### Hedgehog signaling pathway

Analogous to the Hippo pathway, hedgehog predominantly fulfills its function in radiation enteropathy via intricate crosstalk with the WNT or BMP signaling cascades. The hedgehog signaling pathway (Hh) plays a crucial role in regulating various developmental and physiological processes, such as embryonic development, stem cell homeostasis, cell differentiation and proliferation [153–155]. Within the intestinal milieu, Hh ligands operate via a paracrine signaling modality. These ligands, manufactured in the intestinal epithelium, traverse into the mesenchyme to initiate signal transduction, thereby orchestrating diverse mesenchymal cell functions. Moreover, they govern the discharge of WNT and BMP ligands, along with inflammatory mediators, by mesenchymal cells, instituting a feedback loop that, in turn, modulates the IECs themselves [156, 157]. Hh ligands are indispensable for mesenchymal cell multiplication during the genesis of intestinal crypts, which are enveloped by a periphery of fibroblasts proficient in Hh signaling [158, 159]. Suppression of the Hh signaling cascade triggers a marked depletion in the crypt-adjacent fibroblast population, accompanied by a surge in WNT signaling activity within the intestinal epithelium. This perturbation gives rise to a disarray in crypt architecture and impairs the differentiation of intestinal cells, thereby undermining intestinal integrity [160].

#### NF-κB signaling pathway

NF-κB represents a family of transcription factor proteins that are ubiquitous in nearly all animal cells. In orchestrating cellular inflammatory reactions, immune responses, and various other biological processes, NF-κB holds immense significance. However, its dysregulation can potentially instigate autoimmune disorders, chronic inflammation, and a multitude of cancers [161]. In the context of radiation enteropathy, the influence of NF-κB on stem cells is a multifaceted and intricate process. It primarily revolves around inflammation, apoptosis, and cell proliferation. Upon activation after radiation, NF-κB triggers the expression of proinflammatory cytokines such as IL-1β and TNF-α, which can subsequently suppress the proliferation of ISCs [162]. This suppression, in turn, can hinder the timely recovery of the intestinal tissue. Additionally, the activation of NF-κB exerts a profound influence on the intestinal microenvironment, affecting the delicate balance of gut microbiota and immune cells [163, 164]. These alterations can further impact the survival and functional capabilities of stem cells, thereby complicating the already challenging landscape of recovery after radiation. Furthermore, the activation of NF-κB modulates the expression of apoptosis-related genes, like Bax and Bad, thereby promoting stem cell apoptosis. While direct studies investigating the effects of NF-κB on stem cell differentiation are limited, given its crucial role in determining cell fate, its activation may indirectly sway the differentiation trajectory of stem cells towards pathways that are detrimental to intestinal repair in radiation enteropathy. Hence, the NF-κB signaling pathway presents a promising target for developing novel therapeutic approaches to address radiation enteropathy. However, a comprehensive understanding of the underlying mechanisms and the effectiveness of these interventions remains crucial and necessitates extensive further research.

## Potential targets based on other factors

### Metabolic factors

Nutritional alterations have been found to modulate ISCs in the small intestine through diverse metabolic pathways, encompassing oxidative phosphorylation, pyruvate metabolism, fatty acid oxidation, and amino acid metabolism [165–167]. A pivotal player in this regulatory network is Mitochondrial Pyruvate Carrier 1 (MPC1), responsible for shuttling pyruvate into mitochondria. Silencing of MPC1 in Lgr5^+^ ISCs provokes hyperproliferation of these stem cells, illustrating the delicate balance of metabolic regulators [168]. Similarly, knocking down Mitochondrial Chaperone Heat Shock Protein 60 (HSP60) in Lgr5^+^ ISCs disrupts their normal differentiation trajectory, leading to the emergence of aberrant Paneth cells [169]. Carnitine Palmitoyltransferase 1α (CPT1α), a pivotal enzyme in fatty acid oxidation, when specifically suppressed in Lgr5^+^ ISCs, impedes their differentiation progress [170]. Furthermore, 3-Hydroxy-3-Methylglutaryl-CoA Synthase 2 (HMGCS2), a rate-controlling enzyme for ketone body synthesis, also wields influence over ISCs stemness properties. Genetic deletion of HMGCS2 in Lgr5^+^ ISCs diminishes their stemness characteristics, compelling them towards differentiation into Paneth and goblet cell lineages [118]. Keratinocyte Growth Factor (KGF), a nurturing factor for the intestinal epithelium, has demonstrated a salutary impact on intestinal tissues following radiation therapy. Post-irradiation administration of KGF bolsters villus height and crypt depth, alleviating radiotherapy's adverse effects [65] (Table 1). Another enterotrophic agent, Teduglutide, an analogue of Glucagon-like Peptide 2 (GLP-2), likewise safeguards small intestinal stem cells against radiation-induced harm, highlighting the therapeutic potential of metabolic and trophic support in radiation protection and tissue recovery [66] (Table 1).

### Microbiota factors

The intestinal tract teems with a myriad of microorganisms that inhabit the animal's gut, collaboratively facilitating a wide array of physiological and biochemical processes [171–173]. Specifically, *Bifidobacteria* and *Lactobacilli* have been demonstrated, both in vivo and in vitro, to stimulate the proliferation of intestinal Lgr5^+^ ISCs and foster the maturation of the intestinal epithelium, underscoring their probiotic significance [67, 174]. Another study has elucidated the pivotal role of the microbiota in preserving the homeostasis and functionality of ISCs, revealing that 5-hydroxytryptamine (5-HT), a neurotransmitter synthesized by these intestinal microorganisms, fuels ISCs proliferation, further bridging the gap between gut microbiome and stem cell dynamics [67] (Table 1). Moreover, valeric acid (VA), a microbial metabolite produced within the gut, exhibits radioprotective properties [68]. Supplementary intake of VA post-irradiation has been shown to restore the intestinal microflora composition, elevate the survival rates of irradiated mice, shield hematopoietic organs, ameliorate gastrointestinal function, and reinforce the intestinal epithelial integrity in these mice, all without enhancing radioresistance in tumors. These findings highlight the therapeutic potential of gut microbiota modulation and its metabolites in radiation mitigation and tissue restoration [69] (Table 1).

Apart from the well-established molecules known for gut microbiota regulation, a recent groundbreaking report has unveiled a fresh material that remarkably modulates intestinal barrier functionality. The study underscores the efficacy of the HA-β-CD (hyaluronic acid and β-cyclodextrin) supramolecular assembly in suppressing inflammatory reactions, consequently restoring the integrity of the intestinal barrier [70]. Notably, this assembly also exerts a regulatory influence on gut microbiota, marked by a substantial boost in the population of the beneficial probiotic *Akkermansia*, signifying an improved state of intestinal microbiome homeostasis. Collectively, these discoveries point to the promising potential of the HA-based mimetic peptide delivery platform in enhancing both intestinal barrier function and microbiome balance, underscoring its potential application in the treatment of inflammatory bowel disease (IBD). In addition to the utilization of novel materials, recent reports have illuminated the promising prospects of stem cell therapy in addressing radiation-induced intestinal disease. This particular study underscores the remarkable ability of adipose-derived mesenchymal stem cells (ADSC) injections to significantly reverse radiation-induced intestinal damage in living organisms [71]. At the phylum level, the study observed an augmentation of *Bacteroidota* and *Campilobacterota*, coupled with a reduction in *Firmicutes* and *Desulfobacterota*, among irradiated rats. These discoveries emphasize the potential of ADSCs to rectify dysbiosis and reinstate normal colonic flora amidst radiation-induced colonic fibrosis, thereby presenting fresh perspectives for therapeutic strategies aimed at mitigating radiation-induced complications.

## Potential intervention strategies for radiation enteropathy

**Fig. 6:**
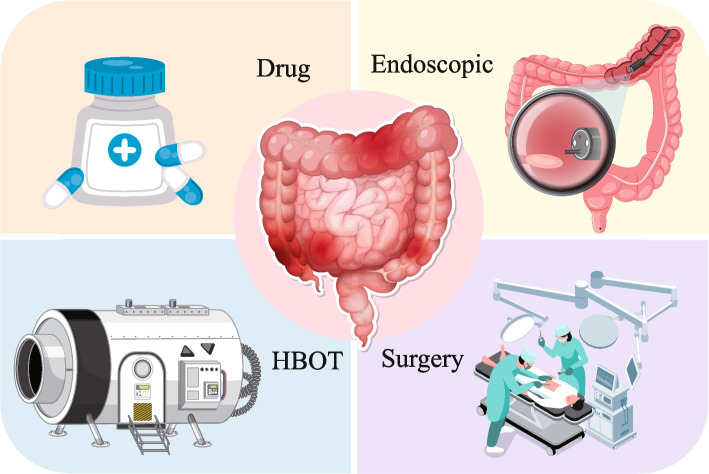
Potential intervention strategies for radiation enteropathy. The principal strategies of treating radiation enteropathy. Pharmacological intervention is up-left, hyperbaric oxygen therapy (HBOT) is down-left, endoscopic procedures is up-right, surgical management is down-right. Figure 6 was modified from Freepik (https://www.freepik.com/)

The current state of treating radiation enteropathy is marked by the coexistence of diverse therapeutic methods, all while posing significant challenges in achieving overall effective outcomes. The principal strategies encompass a comprehensive range, including pharmacological interventions, hyperbaric oxygen therapy, endoscopic procedures, surgical management, each tailored to address the complexity of the condition (Fig. 6).

### Pharmacological interventions

For patients experiencing severe diarrhea, administering antidiarrheal medications can effectively alleviate their symptoms. For those who are sensitive to opioid-based remedies, loperamide or diphenoxylate are viable options, both demonstrating significant efficacy in managing diarrhea stemming from radiation enteropathy [175, 176]. For patients unresponsive to opioid preparations, octreotide, a synthetic long-acting somatostatin analog, may be prescribed [177]. By inhibiting the secretion of vasoactive intestinal peptide, it effectively slows intestinal motility and reduces intestinal fluid and electrolyte production. Furthermore, sucralfate offers a degree of protection against localized mucosal damage, albeit with the caution that it may elevate the risk of intestinal bleeding [178]. Local therapies are also an option, with a 2% benzocaine cottonseed oil retention enema being particularly beneficial for patients experiencing significant tenesmus and pain. Alternatively, hydrocortisone succinate in warm saline can be administered as a retention enema for added relief [179] (Fig. 6).

### Hyperbaric oxygen therapy

Hyperbaric oxygen therapy (HBOT) demonstrates a significant application value and effectiveness in the management of radiation enteropathy [180]. By addressing the root causes of tissue ischemia, hypoxia, and microcirculation failure stemming from vascular endothelial damage, HBOT effectively elevates blood oxygen partial pressure and content [181, 182]. This, in turn, not only mitigates tissue damage but also accelerates ulcer healing and fosters tissue repair processes. Nonetheless, it is imperative to emphasize that HBOT is not a universal solution for all patients with radiation enteropathy. Specifically, it is contraindicated for patients whose condition is in the acute phase or complicated by severe health issues such as intestinal perforation or massive bleeding [183]. Furthermore, during the course of treatment, it is vital to maintain close observation of the patient's response and adhere strictly to the medical professional's guidance to guarantee the safety and efficacy of the therapeutic outcomes (Fig. 6).

### Endoscopic procedures

Endoscopic therapy stands as a pivotal treatment option for radiation enteropathy, renowned for its minimal invasiveness and precision. In recent years, its utilization has surged, incorporating advanced techniques such as laser therapy, argon plasma coagulation (APC), and chemical cauterization [184, 185]. Laser therapy notably curbs intestinal mucosa erosion in radiation proctitis, alleviating persistent bleeding by leveraging laser heat to coagulate damaged tissue. APC, meanwhile, expertly guides argon ion beams to targeted lesions via endoscopy, harnessing ionization-induced thermal energy to coagulate tissue, achieving hemostasis and therapeutic objectives [186]. Endoscopic guidance ensures precise application of formalin solution to bleeding or eroded mucosa, leveraging its chemical cauterizing properties to coagulate and denature proteins, effectively stemming further bleeding [187]. However, it's imperative to emphasize that chemical cauterization necessitates utmost precision to safeguard adjacent healthy tissue. Ultimately, endoscopic therapy occupies a central position in the management of radiation enteropathy, particularly radiation proctitis. Yet, when opting for this therapy, a thorough assessment of patient condition, physical status, and treatment-related risks is imperative to guarantee both treatment safety and efficacy (Fig. 6).

### Surgical management

When confronted with severe manifestations of radiation enteropathy, encompassing massive ulcers, intestinal stenosis stemming from scarring, intestinal obstruction, and intractable massive intestinal bleeding despite non-surgical interventions, it is imperative to tailor surgical approaches based on individual patient conditions. As an illustrative example, for distal colonic lesions, the execution of a transverse colostomy serves as a viable option to facilitate either permanent or temporary fecal diversion, effectively addressing the underlying pathology [184, 185] (Fig. 6).

To summarize, despite the extensive range of treatment options available for radiation enteropathy, there is still no singular, universally safe and effective cure. Consequently, it is of utmost importance to carefully select a personalized treatment plan that precisely addresses each patient's individual condition. Moreover, throughout the course of therapy, it is crucial to closely monitor and address factors such as the patient's nutritional status and psychological wellbeing, with the ultimate goal of optimizing treatment outcomes and enhancing the patient's overall quality of life.

## Conclusions

**Fig. 7:**
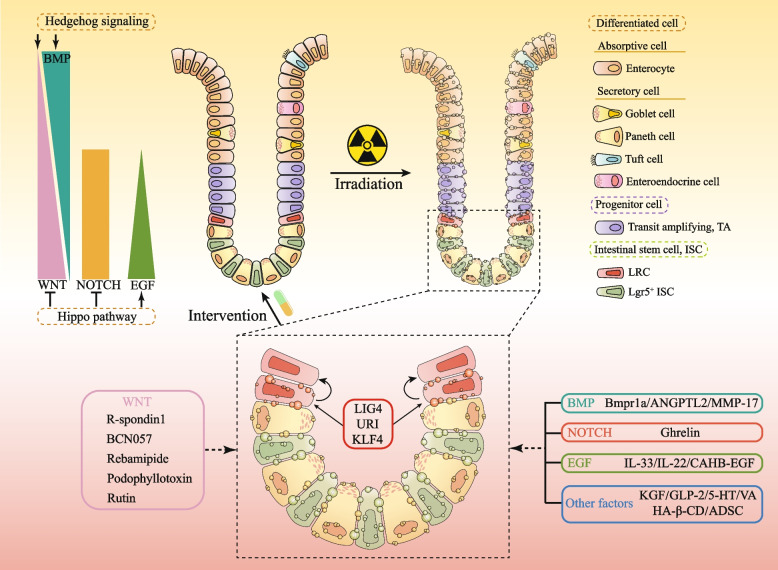
Summary Chart. Main regulatory pathways before intestinal radiation and potential intervention targets after radiation. URI: unconventional prefoldin RPB5 interactor, CA: centella asiatica, TCM: traditional chinese medicine, KGF: keratinocyte growth factor, GLP-2: glucagon-like peptide 2 analogue, 5-HT: 5-hydroxytryptamine, VA: valeric acid, KLF4: Krüppel-like factor 4, HA-β-CD: hyaluronic acid (HA) and β-cyclodextrin (HA-β-CD), ADSC: adipose-derived mesenchymal stem cells, IECs: intestinal epithelial cells, ISCs: intestinal stem cells

Radiotherapy continues to be one of the primary strategies in the management of cancer. However, there has yet to be a significant breakthrough in developing effective strategies to shield normal tissues from radiation damage during therapy. For patients enduring radiotherapy for pelvic, abdominal, or retroperitoneal malignancies, the quest for medications capable of mitigating radiation enteritis and subsequently enhancing their overall quality of life is paramount. In this review, we focused on stem cells, meticulously analyzing the alterations in their key molecular mechanisms and potential therapeutic targets before and after exposure to radiation. This encompassed a thorough examination of shifts in critical pathways such as WNT, BMP, NOTCH, EGF, Hippo, Hedgehog and NF-κB, along with other pertinent factors like nutrient metabolic states and intestinal microbiota. Additionally, we compiled a comprehensive overview of the existing therapeutic strategies for managing radiation enteropathy (Fig. 7). By compiling a comprehensive summary of these underlying mechanisms and treatment methodologies, we aspire to offer some references and insights of innovative therapeutic approaches.

Through this review, it is not difficult for us to find that the current obscurity surrounding the precise pathogenesis of radiation enteropathy presents formidable obstacles to the development of therapeutic agents. While current research has shed light on definitive changes in individual pathways, like the suppression of BMP and the activation of NOTCH and EGF signaling pathways in radiation enteropathy, there still exists ambiguity regarding the behavior of certain pathways, notably WNT. This uncertainty stems from contrasting findings, with some studies indicating its activation while others pointing towards inhibition. Moreover, our prevailing perspective holds that the activation of a specific pathway post-radiation injury, capable of stimulating cell proliferation, is generally deemed advantageous for the repair of small intestinal damage. However, recent findings have highlighted exceptions to this notion. For example, in the URI study, we observed that the overexpression of URI significantly alleviated radiation-induced intestinal symptoms in mice, yet this amelioration was achieved by inhibiting the WNT pathway [53]. Traditionally, activation of the WNT pathway is known to foster cell proliferation. Nevertheless, this research uncovered that excessive activation of the WNT pathway can paradoxically trigger hyperproliferation of ISCs, including LRCs, thereby hindering intestinal damage repair. Consequently, in investigating radiation enteropathy, it is imperative to meticulously balance the degree of activation or inhibition of each pathway, avoiding both extremes as they can yield detrimental outcomes. This underscores one of the complexities in studying radiation enteropathy. Additionally, the intricate interplay and crosstalk among each signaling pathways further complicate the task, rendering the precise delineation of the pathogenesis of radiation enteropathy a lengthy and formidable endeavor.

Furthermore, the pursuit of potential drugs to address radiation enteropathy faces numerous challenges due to the intricate regulatory mechanisms of intestinal stem cells (ISCs) within their distinct microenvironment. Beyond classical signaling pathways, metabolic factors and the complex intestinal microbiota also play pivotal roles in modulating ISCs. While recent mechanistic research has identified several promising agonists or inhibitors that may alleviate radiation enteropathy, it is crucial to consider not only their primary therapeutic effects but also their potential tumor-protective properties, toxicological profiles, and risks of adverse events. Thus, a significant hurdle remains in translating these promising molecules into clinically feasible therapeutic options.

In spite of the intricate and pervasive challenges that accompany the treatment of radiation enteropathy, wherein a definitive and universally effective cure still remains a distant goal, an array of therapeutic modalities is currently at our disposal. These encompass pharmacological interventions, hyperbaric oxygen therapy, endoscopic interventions, and surgical management. The selection of a specific treatment modality is based on the patient's condition and overall health status, complemented by their active participation, positive attitude, and adherence to healthy dietary habits, which all contribute to facilitating recovery. Moving forward, it is imperative that we persist in reinforcing our efforts in fundamental research and clinical trials, relentlessly exploring innovative therapeutic approaches and modalities with the ultimate goal of elevating treatment efficacy and significantly enhancing the quality of life for patients.

## Data Availability

Not applicable.

## References

[1] Hauer-Jensen M, Denham JW, Andreyev HJ. Radiation enteropathy–pathogenesis, treatment and prevention. Nat Rev Gastroenterol Hepatol. 2014;11(8):470–9. 10.1038/nrgastro.2014.46. 24686268 10.1038/nrgastro.2014.46 PMC4346191

[2] Moraitis I, Guiu J, Rubert J. Gut microbiota controlling radiation-induced enteritis and intestinal regeneration. Trends Endocrinol Metab. 2023;34(8):489–501. 10.1016/j.tem.2023.05.006. 37336645 10.1016/j.tem.2023.05.006

[3] Kwak SY, Jang WI, Park S, Cho SS, Lee SB, Kim MJ, et al. Metallothionein 2 activation by pravastatin reinforces epithelial integrity and ameliorates radiation-induced enteropathy. EBioMedicine. 2021;73:103641. 10.1016/j.ebiom.2021.103641. 34688032 10.1016/j.ebiom.2021.103641 PMC8546423

[4] Gandle C, Dhingra S, Agarwal S. Radiation-induced enteritis. Clin Gastroenterol Hepatol. 2020;18(3):A39-a40. 10.1016/j.cgh.2018.11.060. 30529730 10.1016/j.cgh.2018.11.060

[5] Hu B, Jin C, Li HB, Tong J, Ouyang X, Cetinbas NM, et al. The DNA-sensing AIM2 inflammasome controls radiation-induced cell death and tissue injury. Science. 2016;354(6313):765–8. 10.1126/science.aaf7532. 27846608 10.1126/science.aaf7532 PMC5640175

[6] Lenti MV, Di Sabatino A. Intestinal fibrosis. Mol Aspects Med. 2019;65:100–9. 10.1016/j.mam.2018.10.003. 30385174 10.1016/j.mam.2018.10.003

[7] Bachmann R, Heinzelmann F, Müller AC, Ladurner R, Schneider CC, Königsrainer A, et al. Laparoscopic pelvic mesh placement with closure of pelvic floor entrance to prevent small intestine radiation trauma - a retrospective cohort analysis. Int J Surg. 2015;23(Pt A):62–7. 10.1016/j.ijsu.2015.08.082. 26369863 10.1016/j.ijsu.2015.08.082

[8] Chang PY, Qu YQ, Wang J, Dong LH. The potential of mesenchymal stem cells in the management of radiation enteropathy. Cell Death Dis. 2015;6(8):e1840. 10.1038/cddis.2015.189. 26247725 10.1038/cddis.2015.189 PMC4558492

[9] Citrin D, Cotrim AP, Hyodo F, Baum BJ, Krishna MC, Mitchell JB. Radioprotectors and mitigators of radiation-induced normal tissue injury. Oncologist. 2010;15(4):360–71. 10.1634/theoncologist.2009-S104. 20413641 10.1634/theoncologist.2009-S104 PMC3076305

[10] Lam SY, Peppelenbosch MP, Fuhler GM. Prediction and treatment of radiation enteropathy: can intestinal bugs lead the way? Clin Cancer Res. 2019;25(21):6280–2. 10.1158/1078-0432.Ccr-19-2422. 31492747 10.1158/1078-0432.CCR-19-2422

[11] Sato T, Vries RG, Snippert HJ, van de Wetering M, Barker N, Stange DE, et al. Single Lgr5 stem cells build crypt-villus structures in vitro without a mesenchymal niche. Nature. 2009;459(7244):262–5. 10.1038/nature07935. 19329995 10.1038/nature07935

[12] Gehart H, Clevers H. Tales from the crypt: new insights into intestinal stem cells. Nat Rev Gastroenterol Hepatol. 2019;16(1):19–34. 10.1038/s41575-018-0081-y. 30429586 10.1038/s41575-018-0081-y

[13] Kim G, Chen Z, Li J, Luo J, Castro-Martinez F, Wisniewski J, et al. Gut-liver axis calibrates intestinal stem cell fitness. Cell. 2024;187(4):914-30.e20. 10.1016/j.cell.2024.01.001. 38280375 10.1016/j.cell.2024.01.001 PMC10923069

[14] Sato T, van Es JH, Snippert HJ, Stange DE, Vries RG, van den Born M, et al. Paneth cells constitute the niche for Lgr5 stem cells in intestinal crypts. Nature. 2011;469(7330):415–8. 10.1038/nature09637. 21113151 10.1038/nature09637 PMC3547360

[15] Pentinmikko N, Iqbal S, Mana M, Andersson S, Cognetta AB 3rd, Suciu RM, et al. Notum produced by Paneth cells attenuates regeneration of aged intestinal epithelium. Nature. 2019;571(7765):398–402. 10.1038/s41586-019-1383-0. 31292548 10.1038/s41586-019-1383-0 PMC8151802

[16] An Y, Wang C, Fan B, Wang Z, Li Y, Kong F, et al. LSR targets YAP to modulate intestinal Paneth cell differentiation. Cell Rep. 2023;42(9):113118. 10.1016/j.celrep.2023.113118. 37703178 10.1016/j.celrep.2023.113118

[17] Wallaeys C, Garcia-Gonzalez N, Libert C. Paneth cells as the cornerstones of intestinal and organismal health: a primer. EMBO Mol Med. 2023;15(2):e16427. 10.15252/emmm.202216427. 36573340 10.15252/emmm.202216427 PMC9906427

[18] Su X, Jin M, Xu C, Gao Y, Yang Y, Qi H, et al. FABP4 in Paneth cells regulates antimicrobial protein expression to reprogram gut microbiota. Gut Microbes. 2022;14(1):2139978. 10.1080/19490976.2022.2139978. 36519446 10.1080/19490976.2022.2139978 PMC9635462

[19] Qi-Xiang M, Yang F, Ze-Hua H, Nuo-Ming Y, Rui-Long W, Bin-Qiang X, et al. Intestinal TLR4 deletion exacerbates acute pancreatitis through gut microbiota dysbiosis and Paneth cells deficiency. Gut Microbes. 2022;14(1):2112882. 10.1080/19490976.2022.2112882. 35982604 10.1080/19490976.2022.2112882 PMC9397436

[20] Vanuytsel T, Senger S, Fasano A, Shea-Donohue T. Major signaling pathways in intestinal stem cells. Biochim Biophys Acta. 2013;1830(2):2410–26. 10.1016/j.bbagen.2012.08.006. 22922290 10.1016/j.bbagen.2012.08.006 PMC4408610

[21] Clevers H. The intestinal crypt, a prototype stem cell compartment. Cell. 2013;154(2):274–84. 10.1016/j.cell.2013.07.004. 23870119 10.1016/j.cell.2013.07.004

[22] Cheung P, Xiol J, Dill MT, Yuan WC, Panero R, Roper J, et al. Regenerative reprogramming of the intestinal stem cell state via Hippo signaling suppresses metastatic colorectal cancer. Cell Stem Cell. 2020;27(4):590-604.e9. 10.1016/j.stem.2020.07.003. 32730753 10.1016/j.stem.2020.07.003 PMC10114498

[23] Hinshaw DC, Swain CA, Chen D, Hanna A, Molina PA, Maynard CL, et al. Hedgehog blockade remodels the gut microbiota and the intestinal effector CD8(+) T cells in a mouse model of mammary carcinoma. Lab Invest. 2022;102(11):1236–44. 10.1038/s41374-022-00828-1. 35907952 10.1038/s41374-022-00828-1 PMC13180488

[24] Booth C, Tudor G, Tudor J, Katz BP, MacVittie TJ. Acute gastrointestinal syndrome in high-dose irradiated mice. Health Phys. 2012;103(4):383–99. 10.1097/hp.0b013e318266ee13. 23091876 10.1097/hp.0b013e318266ee13 PMC3530834

[25] Bao W, You Y, Ni J, Hou H, Lyu J, Feng G, et al. Inhibiting sorting nexin 10 promotes mucosal healing through SREBP2-mediated stemness restoration of intestinal stem cells. Sci Adv. 2023;9(35):eadh5016. 10.1126/sciadv.adh5016. 37647408 10.1126/sciadv.adh5016 PMC10468130

[26] Leibowitz BJ, Zhao G, Wei L, Ruan H, Epperly M, Chen L, et al. Interferon b drives intestinal regeneration after radiation. Sci Adv. 2021;7(41):eabi5253. 10.1126/sciadv.abi5253. 34613772 10.1126/sciadv.abi5253 PMC8494436

[27] Sanman LE, Chen IW, Bieber JM, Steri V, Trentesaux C, Hann B, et al. Transit-amplifying cells coordinate changes in intestinal epithelial cell-type composition. Dev Cell. 2021;56(3):356-65.e9. 10.1016/j.devcel.2020.12.020. 33484640 10.1016/j.devcel.2020.12.020 PMC7917018

[28] Jiang Z, Wu F, Laise P, Takayuki T, Na F, Kim W, et al. Tff2 defines transit-amplifying pancreatic acinar progenitors that lack regenerative potential and are protective against Kras-driven carcinogenesis. Cell Stem Cell. 2023;30(8):1091-109.e7. 10.1016/j.stem.2023.07.002. 37541213 10.1016/j.stem.2023.07.002 PMC10414754

[29] Sun Q, Lee W, Hu H, Ogawa T, De Leon S, Katehis I, et al. Dedifferentiation maintains melanocyte stem cells in a dynamic niche. Nature. 2023;616(7958):774–82. 10.1038/s41586-023-05960-6. 37076619 10.1038/s41586-023-05960-6 PMC10132989

[30] Chaves-Pérez A, Santos-de-Frutos K, de la Rosa S, Herranz-Montoya I, Perna C, Djouder N. Transit-amplifying cells control R-spondins in the mouse crypt to modulate intestinal stem cell proliferation. J Exp Med. 2022;219(11). 10.1084/jem.20212405. 10.1084/jem.20212405 PMC9475298 36098959

[31] Fawkner-Corbett D, Antanaviciute A, Parikh K, Jagielowicz M, Gerós AS, Gupta T, et al. Spatiotemporal analysis of human intestinal development at single-cell resolution. Cell. 2021;184(3):810-26.e23. 10.1016/j.cell.2020.12.016. 33406409 10.1016/j.cell.2020.12.016 PMC7864098

[32] Goto N, Goto S, Imada S, Hosseini S, Deshpande V, Yilmaz ÖH. Lymphatics and fibroblasts support intestinal stem cells in homeostasis and injury. Cell Stem Cell. 2022;29(8):1246-61.e6. 10.1016/j.stem.2022.06.013. 35931033 10.1016/j.stem.2022.06.013 PMC9720889

[33] Gu J, Chen YZ, Zhang ZX, Yang ZX, Duan GX, Qin LQ, et al. At what dose can total body and whole abdominal irradiation cause lethal intestinal injury among C57BL/6J mice? Dose Response. 2020;18(3):1559325820956783. 10.1177/1559325820956783. 32973418 10.1177/1559325820956783 PMC7493248

[34] Metcalfe C, Kljavin NM, Ybarra R, de Sauvage FJ. Lgr5+ stem cells are indispensable for radiation-induced intestinal regeneration. Cell Stem Cell. 2014;14(2):149–59. 10.1016/j.stem.2013.11.008. 24332836 10.1016/j.stem.2013.11.008

[35] Bjerknes M, Cheng H. Clonal analysis of mouse intestinal epithelial progenitors. Gastroenterology. 1999;116(1):7–14. 10.1016/s0016-5085(99)70222-2. 9869596 10.1016/s0016-5085(99)70222-2

[36] Ludikhuize MC, Meerlo M, Gallego MP, Xanthakis D, Burgaya Julià M, Nguyen NTB, et al. Mitochondria define intestinal stem cell differentiation downstream of a FOXO/Notch axis. Cell Metab. 2020;32(5):889-900.e7. 10.1016/j.cmet.2020.10.005. 33147486 10.1016/j.cmet.2020.10.005

[37] Scoville DH, Sato T, He XC, Li L. Current view: intestinal stem cells and signaling. Gastroenterology. 2008;134(3):849–64. 10.1053/j.gastro.2008.01.079. 18325394 10.1053/j.gastro.2008.01.079

[38] Barker N, van Es JH, Kuipers J, Kujala P, van den Born M, Cozijnsen M, et al. Identification of stem cells in small intestine and colon by marker gene Lgr5. Nature. 2007;449(7165):1003–7. 10.1038/nature06196. 17934449 10.1038/nature06196

[39] Potten CS, Hume WJ, Reid P, Cairns J. The segregation of DNA in epithelial stem cells. Cell. 1978;15(3):899–906. 10.1016/0092-8674(78)90274-x. 728994 10.1016/0092-8674(78)90274-x

[40] Tsai YH, VanDussen KL, Sawey ET, Wade AW, Kasper C, Rakshit S, et al. ADAM10 regulates Notch function in intestinal stem cells of mice. Gastroenterology. 2014;147(4):822-34.e13. 10.1053/j.gastro.2014.07.003. 25038433 10.1053/j.gastro.2014.07.003 PMC4176890

[41] Sangiorgi E, Capecchi MR. Bmi1 is expressed in vivo in intestinal stem cells. Nat Genet. 2008;40(7):915–20. 10.1038/ng.165. 18536716 10.1038/ng.165 PMC2906135

[42] Breault DT, Min IM, Carlone DL, Farilla LG, Ambruzs DM, Henderson DE, et al. Generation of mTert-GFP mice as a model to identify and study tissue progenitor cells. Proc Natl Acad Sci U S A. 2008;105(30):10420–5. 10.1073/pnas.0804800105. 18650388 10.1073/pnas.0804800105 PMC2492454

[43] Takeda N, Jain R, LeBoeuf MR, Wang Q, Lu MM, Epstein JA. Interconversion between intestinal stem cell populations in distinct niches. Science. 2011;334(6061):1420–4. 10.1126/science.1213214. 22075725 10.1126/science.1213214 PMC3705713

[44] Powell AE, Wang Y, Li Y, Poulin EJ, Means AL, Washington MK, et al. The pan-ErbB negative regulator Lrig1 is an intestinal stem cell marker that functions as a tumor suppressor. Cell. 2012;149(1):146–58. 10.1016/j.cell.2012.02.042. 22464327 10.1016/j.cell.2012.02.042 PMC3563328

[45] Muñoz J, Stange DE, Schepers AG, van de Wetering M, Koo BK, Itzkovitz S, et al. The Lgr5 intestinal stem cell signature: robust expression of proposed quiescent “+4” cell markers. Embo j. 2012;31(14):3079–91. 10.1038/emboj.2012.166. 22692129 10.1038/emboj.2012.166 PMC3400017

[46] Yan KS, Chia LA, Li X, Ootani A, Su J, Lee JY, et al. The intestinal stem cell markers Bmi1 and Lgr5 identify two functionally distinct populations. Proc Natl Acad Sci U S A. 2012;109(2):466–71. 10.1073/pnas.1118857109. 22190486 10.1073/pnas.1118857109 PMC3258636

[47] Kim CK, Yang VW, Bialkowska AB. The role of intestinal stem cells in epithelial regeneration following radiation-induced gut injury. Curr Stem Cell Rep. 2017;3(4):320–32. 10.1007/s40778-017-0103-7. 29497599 10.1007/s40778-017-0103-7 PMC5818549

[48] Hua G, Thin TH, Feldman R, Haimovitz-Friedman A, Clevers H, Fuks Z, et al. Crypt base columnar stem cells in small intestines of mice are radioresistant. Gastroenterology. 2012;143(5):1266–76. 10.1053/j.gastro.2012.07.106. 22841781 10.1053/j.gastro.2012.07.106 PMC3480544

[49] Beumer J, Clevers H. Regulation and plasticity of intestinal stem cells during homeostasis and regeneration. Development. 2016;143(20):3639–49. 10.1242/dev.133132. 27802133 10.1242/dev.133132

[50] Tian H, Biehs B, Warming S, Leong KG, Rangell L, Klein OD, et al. A reserve stem cell population in small intestine renders Lgr5-positive cells dispensable. Nature. 2011;478(7368):255–9. 10.1038/nature10408. 21927002 10.1038/nature10408 PMC4251967

[51] Bhanja P, Saha S, Kabarriti R, Liu L, Roy-Chowdhury N, Roy-Chowdhury J, et al. Protective role of R-spondin1, an intestinal stem cell growth factor, against radiation-induced gastrointestinal syndrome in mice. PLoS ONE. 2009;4(11):e8014. 10.1371/journal.pone.0008014. 19956666 10.1371/journal.pone.0008014 PMC2777375

[52] Jun S, Jung YS, Suh HN, Wang W, Kim MJ, Oh YS, et al. LIG4 mediates Wnt signalling-induced radioresistance. Nat Commun. 2016;7:10994. 10.1038/ncomms10994. 27009971 10.1038/ncomms10994 PMC4820809

[53] Chaves-Pérez A, Yilmaz M, Perna C, de la Rosa S, Djouder N. URI is required to maintain intestinal architecture during ionizing radiation. Science. 2019;364(6443):10.1126/science.aaq1165. 10.1126/science.aaq1165 31147493

[54] Bhanja P, Norris A, Gupta-Saraf P, Hoover A, Saha S. BCN057 induces intestinal stem cell repair and mitigates radiation-induced intestinal injury. Stem Cell Res Ther. 2018;9(1):26. 10.1186/s13287-017-0763-3. 29394953 10.1186/s13287-017-0763-3 PMC5797353

[55] Shim S, Jang HS, Myung HW, Myung JK, Kang JK, Kim MJ, et al. Rebamipide ameliorates radiation-induced intestinal injury in a mouse model. Toxicol Appl Pharmacol. 2017;329:40–7. 10.1016/j.taap.2017.05.012. 28526636 10.1016/j.taap.2017.05.012

[56] Kalita B, Ranjan R, Gupta ML. Combination treatment of podophyllotoxin and rutin promotes mouse Lgr5(+ ve) intestinal stem cells survival against lethal radiation injury through Wnt signaling. Apoptosis. 2019;24(3–4):326–40. 10.1007/s10495-019-01519-x. 30725351 10.1007/s10495-019-01519-x

[57] Qi Z, Li Y, Zhao B, Xu C, Liu Y, Li H, et al. BMP restricts stemness of intestinal Lgr5(+) stem cells by directly suppressing their signature genes. Nat Commun. 2017;8:13824. 10.1038/ncomms13824. 28059064 10.1038/ncomms13824 PMC5227110

[58] Horiguchi H, Endo M, Kawane K, Kadomatsu T, Terada K, Morinaga J, et al. ANGPTL2 expression in the intestinal stem cell niche controls epithelial regeneration and homeostasis. Embo j. 2017;36(4):409–24. 10.15252/embj.201695690. 28043948 10.15252/embj.201695690 PMC5694950

[59] Martín-Alonso M, Iqbal S, Vornewald PM, Lindholm HT, Damen MJ, Martínez F, et al. Smooth muscle-specific MMP17 (MT4-MMP) regulates the intestinal stem cell niche and regeneration after damage. Nat Commun. 2021;12(1):6741. 10.1038/s41467-021-26904-6. 34795242 10.1038/s41467-021-26904-6 PMC8602650

[60] Kwak SY, Shim S, Park S, Kim H, Lee SJ, Kim MJ, et al. Ghrelin reverts intestinal stem cell loss associated with radiation-induced enteropathy by activating Notch signaling. Phytomedicine. 2021;81:153424. 10.1016/j.phymed.2020.153424. 33278782 10.1016/j.phymed.2020.153424

[61] Calafiore M, Fu YY, Vinci P, Arnhold V, Chang WY, Jansen SA, et al. A tissue-intrinsic IL-33/EGF circuit promotes epithelial regeneration after intestinal injury. Nat Commun. 2023;14(1):5411. 10.1038/s41467-023-40993-5. 37669929 10.1038/s41467-023-40993-5 PMC10480426

[62] Lindemans CA, Calafiore M, Mertelsmann AM, O’Connor MH, Dudakov JA, Jenq RR, et al. Interleukin-22 promotes intestinal-stem-cell-mediated epithelial regeneration. Nature. 2015;528(7583):560–4. 10.1038/nature16460. 26649819 10.1038/nature16460 PMC4720437

[63] Matthews MA, Watkins D, Darbyshire A, Carson WE, Besner GE. Heparin-binding EGF-like growth factor (HB-EGF) protects the intestines from radiation therapy-induced intestinal injury. J Pediatr Surg. 2013;48(6):1316–22. 10.1016/j.jpedsurg.2013.03.030. 23845625 10.1016/j.jpedsurg.2013.03.030 PMC3710435

[64] Kwak SY, Jang WI, Lee SB, Kim MJ, Park S, Cho SS, et al. Centella asiatica-derived endothelial paracrine restores epithelial barrier dysfunction in radiation-induced enteritis. Cells. 2022;11(16). 10.3390/cells11162544. 10.3390/cells11162544 PMC9406831 36010621

[65] Farrell CL, Bready JV, Rex KL, Chen JN, DiPalma CR, Whitcomb KL, et al. Keratinocyte growth factor protects mice from chemotherapy and radiation-induced gastrointestinal injury and mortality. Cancer Res. 1998;58(5):933–9. 9500453

[66] Booth C, Booth D, Williamson S, Demchyshyn LL, Potten CS. Teduglutide ([Gly2]GLP-2) protects small intestinal stem cells from radiation damage. Cell Prolif. 2004;37(6):385–400. 10.1111/j.1365-2184.2004.00320.x. 15548172 10.1111/j.1365-2184.2004.00320.x PMC6495530

[67] Zhu P, Lu T, Wu J, Fan D, Liu B, Zhu X, et al. Gut microbiota drives macrophage-dependent self-renewal of intestinal stem cells via niche enteric serotonergic neurons. Cell Res. 2022;32(6):555–69. 10.1038/s41422-022-00645-7. 35379903 10.1038/s41422-022-00645-7 PMC9160288

[68] Yuille S, Reichardt N, Panda S, Dunbar H, Mulder IE. Human gut bacteria as potent class I histone deacetylase inhibitors in vitro through production of butyric acid and valeric acid. PLoS ONE. 2018;13(7):e0201073. 10.1371/journal.pone.0201073. 30052654 10.1371/journal.pone.0201073 PMC6063406

[69] Li Y, Dong J, Xiao H, Zhang S, Wang B, Cui M, et al. Gut commensal derived-valeric acid protects against radiation injuries. Gut Microbes. 2020;11(4):789–806. 10.1080/19490976.2019.1709387. 31931652 10.1080/19490976.2019.1709387 PMC7524389

[70] Fu E, Qian M, He N, Yin Y, Liu Y, Han Z, et al. Biomimetic Supramolecular Assembly with IGF-1C Delivery Ameliorates Inflammatory Bowel Disease (IBD) by Restoring Intestinal Barrier Integrity. Adv Sci (Weinh). 2024;e2403075. 10.1002/advs.202403075. 10.1002/advs.202403075 PMC11423171 39041890

[71] Thandar M, Yang X, Zhu Y, Zhang X, Chen Z, Huang S, et al. Dysbiosis of gut microbiota and metabolites is associated with radiation-induced colorectal fibrosis and is restored by adipose-derived mesenchymal stem cell therapy. Life Sci. 2024;341:122502. 10.1016/j.lfs.2024.122502. 38350495 10.1016/j.lfs.2024.122502

[72] Yan KS, Janda CY, Chang J, Zheng GXY, Larkin KA, Luca VC, et al. Non-equivalence of Wnt and R-spondin ligands during Lgr5(+) intestinal stem-cell self-renewal. Nature. 2017;545(7653):238–42. 10.1038/nature22313. 28467820 10.1038/nature22313 PMC5641471

[73] Li Y, Chen M, Hu J, Sheng R, Lin Q, He X, et al. Volumetric compression induces intracellular crowding to control intestinal organoid growth via Wnt/β-Catenin signaling. Cell Stem Cell. 2021;28(1):63-78.e7. 10.1016/j.stem.2020.09.012. 33053374 10.1016/j.stem.2020.09.012 PMC7796961

[74] Hao HX, Xie Y, Zhang Y, Charlat O, Oster E, Avello M, et al. ZNRF3 promotes Wnt receptor turnover in an R-spondin-sensitive manner. Nature. 2012;485(7397):195–200. 10.1038/nature11019. 22575959 10.1038/nature11019

[75] Koo BK, Spit M, Jordens I, Low TY, Stange DE, van de Wetering M, et al. Tumour suppressor RNF43 is a stem-cell E3 ligase that induces endocytosis of Wnt receptors. Nature. 2012;488(7413):665–9. 10.1038/nature11308. 22895187 10.1038/nature11308

[76] Liu J, Xiao Q, Xiao J, Niu C, Li Y, Zhang X, et al. Wnt/β-catenin signalling: function, biological mechanisms, and therapeutic opportunities. Signal Transduct Target Ther. 2022;7(1):3. 10.1038/s41392-021-00762-6. 34980884 10.1038/s41392-021-00762-6 PMC8724284

[77] Mah AT, Yan KS, Kuo CJ. Wnt pathway regulation of intestinal stem cells. J Physiol. 2016;594(17):4837–47. 10.1113/jp271754. 27581568 10.1113/JP271754 PMC5009769

[78] Boonekamp KE, Heo I, Artegiani B, Asra P, van Son G, de Ligt J, et al. Identification of novel human Wnt target genes using adult endodermal tissue-derived organoids. Dev Biol. 2021;474:37–47. 10.1016/j.ydbio.2021.01.009. 33571486 10.1016/j.ydbio.2021.01.009

[79] Lu Q, Yang D, Li H, Niu T, Tong A. Multiple myeloma: signaling pathways and targeted therapy. Mol Biomed. 2024;5(1):25. 10.1186/s43556-024-00188-w. 38961036 10.1186/s43556-024-00188-w PMC11222366

[80] Pinto D, Gregorieff A, Begthel H, Clevers H. Canonical Wnt signals are essential for homeostasis of the intestinal epithelium. Genes Dev. 2003;17(14):1709–13. 10.1101/gad.267103. 12865297 10.1101/gad.267103 PMC196179

[81] Fevr T, Robine S, Louvard D, Huelsken J. Wnt/beta-catenin is essential for intestinal homeostasis and maintenance of intestinal stem cells. Mol Cell Biol. 2007;27(21):7551–9. 10.1128/mcb.01034-07. 17785439 10.1128/MCB.01034-07 PMC2169070

[82] Muncan V, Sansom OJ, Tertoolen L, Phesse TJ, Begthel H, Sancho E, et al. Rapid loss of intestinal crypts upon conditional deletion of the Wnt/Tcf-4 target gene c-Myc. Mol Cell Biol. 2006;26(22):8418–26. 10.1128/mcb.00821-06. 16954380 10.1128/MCB.00821-06 PMC1636776

[83] Korinek V, Barker N, Moerer P, van Donselaar E, Huls G, Peters PJ, et al. Depletion of epithelial stem-cell compartments in the small intestine of mice lacking Tcf-4. Nat Genet. 1998;19(4):379–83. 10.1038/1270. 9697701 10.1038/1270

[84] Kim KA, Kakitani M, Zhao J, Oshima T, Tang T, Binnerts M, et al. Mitogenic influence of human R-spondin1 on the intestinal epithelium. Science. 2005;309(5738):1256–9. 10.1126/science.1112521. 16109882 10.1126/science.1112521

[85] Sansom OJ, Reed KR, Hayes AJ, Ireland H, Brinkmann H, Newton IP, et al. Loss of Apc in vivo immediately perturbs Wnt signaling, differentiation, and migration. Genes Dev. 2004;18(12):1385–90. 10.1101/gad.287404. 15198980 10.1101/gad.287404 PMC423189

[86] Barker N, Ridgway RA, van Es JH, van de Wetering M, Begthel H, van den Born M, et al. Crypt stem cells as the cells-of-origin of intestinal cancer. Nature. 2009;457(7229):608–11. 10.1038/nature07602. 19092804 10.1038/nature07602

[87] Kouvaris JR, Kouloulias VE, Vlahos LJ. Amifostine: the first selective-target and broad-spectrum radioprotector. Oncologist. 2007;12(6):738–47. 10.1634/theoncologist.12-6-738. 17602063 10.1634/theoncologist.12-6-738

[88] Wang S, Chen YG. BMP signaling in homeostasis, transformation and inflammatory response of intestinal epithelium. Sci China Life Sci. 2018;61(7):800–7. 10.1007/s11427-018-9310-7. 29855793 10.1007/s11427-018-9310-7

[89] Tie Y, Tang F, Peng D, Zhang Y, Shi H. TGF-beta signal transduction: biology, function and therapy for diseases. Mol Biomed. 2022;3(1):45. 10.1186/s43556-022-00109-9. 36534225 10.1186/s43556-022-00109-9 PMC9761655

[90] Lowery JW, Rosen V. The BMP Pathway and Its Inhibitors in the Skeleton. Physiol Rev. 2018;98(4):2431–52. 10.1152/physrev.00028.2017. 30156494 10.1152/physrev.00028.2017

[91] Zhang Y, Que J. BMP signaling in development, stem cells, and diseases of the gastrointestinal tract. Annu Rev Physiol. 2020;82:251–73. 10.1146/annurev-physiol-021119-034500. 31618602 10.1146/annurev-physiol-021119-034500 PMC13090088

[92] Wu M, Chen G, Li YP. TGF-β and BMP signaling in osteoblast, skeletal development, and bone formation, homeostasis and disease. Bone Res. 2016;4:16009. 10.1038/boneres.2016.9. 27563484 10.1038/boneres.2016.9 PMC4985055

[93] Heldin CH, Miyazono K, ten Dijke P. TGF-beta signalling from cell membrane to nucleus through SMAD proteins. Nature. 1997;390(6659):465–71. 10.1038/37284. 9393997 10.1038/37284

[94] Jaswal AP, Kumar B, Roelofs AJ, Iqbal SF, Singh AK, Riemen AHK, et al. BMP signaling: a significant player and therapeutic target for osteoarthritis. Osteoarthritis Cartilage. 2023;31(11):1454–68. 10.1016/j.joca.2023.05.016. 37392862 10.1016/j.joca.2023.05.016

[95] Alberici P, Jagmohan-Changur S, De Pater E, Van Der Valk M, Smits R, Hohenstein P, et al. Smad4 haploinsufficiency in mouse models for intestinal cancer. Oncogene. 2006;25(13):1841–51. 10.1038/sj.onc.1209226. 16288217 10.1038/sj.onc.1209226

[96] Chen G, Xu H, Yao Y, Xu T, Yuan M, Zhang X, et al. BMP signaling in the development and regeneration of cranium bones and maintenance of calvarial stem cells. Front Cell Dev Biol. 2020;8:135. 10.3389/fcell.2020.00135. 32211409 10.3389/fcell.2020.00135 PMC7075941

[97] Voorneveld PW, Kodach LL, Jacobs RJ, Liv N, Zonnevylle AC, Hoogenboom JP, et al. Loss of SMAD4 alters BMP signaling to promote colorectal cancer cell metastasis via activation of Rho and ROCK. Gastroenterology. 2014;147(1):196-208.e13. 10.1053/j.gastro.2014.03.052. 24704720 10.1053/j.gastro.2014.03.052

[98] Yum MK, Han S, Fink J, Wu SS, Dabrowska C, Trendafilova T, et al. Tracing oncogene-driven remodelling of the intestinal stem cell niche. Nature. 2021;594(7863):442–7. 10.1038/s41586-021-03605-0. 34079126 10.1038/s41586-021-03605-0 PMC7614896

[99] Kosinski C, Li VS, Chan AS, Zhang J, Ho C, Tsui WY, et al. Gene expression patterns of human colon tops and basal crypts and BMP antagonists as intestinal stem cell niche factors. Proc Natl Acad Sci U S A. 2007;104(39):15418–23. 10.1073/pnas.0707210104. 17881565 10.1073/pnas.0707210104 PMC2000506

[100] Henríquez JP, Krull CE, Osses N. The Wnt and BMP families of signaling morphogens at the vertebrate neuromuscular junction. Int J Mol Sci. 2011;12(12):8924–46. 10.3390/ijms12128924. 22272112 10.3390/ijms12128924 PMC3257109

[101] Haramis AP, Begthel H, van den Born M, van Es J, Jonkheer S, Offerhaus GJ, et al. De novo crypt formation and juvenile polyposis on BMP inhibition in mouse intestine. Science. 2004;303(5664):1684–6. 10.1126/science.1093587. 15017003 10.1126/science.1093587

[102] Davis H, Irshad S, Bansal M, Rafferty H, Boitsova T, Bardella C, et al. Aberrant epithelial GREM1 expression initiates colonic tumorigenesis from cells outside the stem cell niche. Nat Med. 2015;21(1):62–70. 10.1038/nm.3750. 25419707 10.1038/nm.3750 PMC4594755

[103] He XC, Zhang J, Tong WG, Tawfik O, Ross J, Scoville DH, et al. BMP signaling inhibits intestinal stem cell self-renewal through suppression of Wnt-beta-catenin signaling. Nat Genet. 2004;36(10):1117–21. 10.1038/ng1430. 15378062 10.1038/ng1430

[104] Kraiczy J, McCarthy N, Malagola E, Tie G, Madha S, Boffelli D, et al. Graded BMP signaling within intestinal crypt architecture directs self-organization of the Wnt-secreting stem cell niche. Cell Stem Cell. 2023;30(4):433-49.e8. 10.1016/j.stem.2023.03.004. 37028407 10.1016/j.stem.2023.03.004 PMC10134073

[105] Demitrack ES, Samuelson LC. Notch regulation of gastrointestinal stem cells. J Physiol. 2016;594(17):4791–803. 10.1113/jp271667. 26848053 10.1113/JP271667 PMC5009795

[106] Schröder N, Gossler A. Expression of Notch pathway components in fetal and adult mouse small intestine. Gene Expr Patterns. 2002;2(3–4):247–50. 10.1016/s1567-133x(02)00060-1. 12617809 10.1016/s1567-133x(02)00060-1

[107] Zhou B, Lin W, Long Y, Yang Y, Zhang H, Wu K, et al. Notch signaling pathway: architecture, disease, and therapeutics. Signal Transduct Target Ther. 2022;7(1):95. 10.1038/s41392-022-00934-y. 35332121 10.1038/s41392-022-00934-y PMC8948217

[108] Gjorevski N, Nikolaev M, Brown TE, Mitrofanova O, Brandenberg N, DelRio FW, et al. Tissue geometry drives deterministic organoid patterning. Science. 2022;375(6576):eaaw9021. 10.1126/science.aaw9021. 34990240 10.1126/science.aaw9021 PMC9131435

[109] Kunze B, Wein F, Fang HY, Anand A, Baumeister T, Strangmann J, et al. Notch signaling mediates differentiation in barrett’s esophagus and promotes progression to adenocarcinoma. Gastroenterology. 2020;159(2):575–90. 10.1053/j.gastro.2020.04.033. 32325086 10.1053/j.gastro.2020.04.033 PMC7484392

[110] Siebel C, Lendahl U. Notch signaling in development, tissue homeostasis, and disease. Physiol Rev. 2017;97(4):1235–94. 10.1152/physrev.00005.2017. 28794168 10.1152/physrev.00005.2017

[111] Xia R, Xu M, Yang J, Ma X. The role of Hedgehog and Notch signaling pathway in cancer. Mol Biomed. 2022;3(1):44. 10.1186/s43556-022-00099-8. 36517618 10.1186/s43556-022-00099-8 PMC9751255

[112] Krishnamurthy N, Kurzrock R. Targeting the Wnt/beta-catenin pathway in cancer: update on effectors and inhibitors. Cancer Treat Rev. 2018;62:50–60. 10.1016/j.ctrv.2017.11.002. 29169144 10.1016/j.ctrv.2017.11.002 PMC5745276

[113] Fre S, Hannezo E, Sale S, Huyghe M, Lafkas D, Kissel H, et al. Notch lineages and activity in intestinal stem cells determined by a new set of knock-in mice. PLoS ONE. 2011;6(10):e25785. 10.1371/journal.pone.0025785. 21991352 10.1371/journal.pone.0025785 PMC3185035

[114] Bohin N, Keeley TM, Carulli AJ, Walker EM, Carlson EA, Gao J, et al. Rapid crypt cell remodeling regenerates the intestinal stem cell niche after Notch inhibition. Stem Cell Reports. 2020;15(1):156–70. 10.1016/j.stemcr.2020.05.010. 32531190 10.1016/j.stemcr.2020.05.010 PMC7363878

[115] Riccio O, van Gijn ME, Bezdek AC, Pellegrinet L, van Es JH, Zimber-Strobl U, et al. Loss of intestinal crypt progenitor cells owing to inactivation of both Notch1 and Notch2 is accompanied by derepression of CDK inhibitors p27Kip1 and p57Kip2. EMBO Rep. 2008;9(4):377–83. 10.1038/embor.2008.7. 18274550 10.1038/embor.2008.7 PMC2288761

[116] Nauman M, Stanley P. Glycans that regulate Notch signaling in the intestine. Biochem Soc Trans. 2022;50(2):689–701. 10.1042/bst20200782. 35311893 10.1042/BST20200782 PMC9370068

[117] Shroyer NF, Helmrath MA, Wang VY, Antalffy B, Henning SJ, Zoghbi HY. Intestine-specific ablation of mouse atonal homolog 1 (Math1) reveals a role in cellular homeostasis. Gastroenterology. 2007;132(7):2478–88. 10.1053/j.gastro.2007.03.047. 17570220 10.1053/j.gastro.2007.03.047

[118] Cheng CW, Biton M, Haber AL, Gunduz N, Eng G, Gaynor LT, et al. Ketone Body Signaling Mediates Intestinal Stem Cell Homeostasis and Adaptation to Diet. Cell. 2019;178(5):1115-31.e15. 10.1016/j.cell.2019.07.048. 31442404 10.1016/j.cell.2019.07.048 PMC6732196

[119] van Es JH, van Gijn ME, Riccio O, van den Born M, Vooijs M, Begthel H, et al. Notch/gamma-secretase inhibition turns proliferative cells in intestinal crypts and adenomas into goblet cells. Nature. 2005;435(7044):959–63. 10.1038/nature03659. 15959515 10.1038/nature03659

[120] VanDussen KL, Carulli AJ, Keeley TM, Patel SR, Puthoff BJ, Magness ST, et al. Notch signaling modulates proliferation and differentiation of intestinal crypt base columnar stem cells. Development. 2012;139(3):488–97. 10.1242/dev.070763. 22190634 10.1242/dev.070763 PMC3252352

[121] Fre S, Huyghe M, Mourikis P, Robine S, Louvard D, Artavanis-Tsakonas S. Notch signals control the fate of immature progenitor cells in the intestine. Nature. 2005;435(7044):964–8. 10.1038/nature03589. 15959516 10.1038/nature03589

[122] Pope JL, Bhat AA, Sharma A, Ahmad R, Krishnan M, Washington MK, et al. Claudin-1 regulates intestinal epithelial homeostasis through the modulation of Notch-signalling. Gut. 2014;63(4):622–34. 10.1136/gutjnl-2012-304241. 23766441 10.1136/gutjnl-2012-304241 PMC4083824

[123] Kadur Lakshminarasimha Murthy P, Srinivasan T, Bochter MS, Xi R, Varanko AK, Tung KL, et al. Radical and lunatic fringes modulate notch ligands to support mammalian intestinal homeostasis. Elife. 2018;7(10.7554/eLife.35710. 10.7554/eLife.35710 PMC5896954 29629872

[124] Hageman JH, Heinz MC, Kretzschmar K, van der Vaart J, Clevers H, Snippert HJG. Intestinal regeneration: regulation by the microenvironment. Dev Cell. 2020;54(4):435–46. 10.1016/j.devcel.2020.07.009. 32841594 10.1016/j.devcel.2020.07.009

[125] Ishibashi F, Shimizu H, Nakata T, Fujii S, Suzuki K, Kawamoto A, et al. Contribution of ATOH1(+) Cells to the homeostasis, repair, and tumorigenesis of the colonic epithelium. Stem Cell Reports. 2018;10(1):27–42. 10.1016/j.stemcr.2017.11.006. 29233556 10.1016/j.stemcr.2017.11.006 PMC5768891

[126] Yu S, Tong K, Zhao Y, Balasubramanian I, Yap GS, Ferraris RP, et al. Paneth cell multipotency induced by Notch activation following injury. Cell Stem Cell. 2018;23(1):46-59.e5. 10.1016/j.stem.2018.05.002. 29887318 10.1016/j.stem.2018.05.002 PMC6035085

[127] Qu D, May R, Sureban SM, Weygant N, Chandrakesan P, Ali N, et al. Inhibition of Notch signaling reduces the number of surviving Dclk1+ reserve crypt epithelial stem cells following radiation injury. Am J Physiol Gastrointest Liver Physiol. 2014;306(5):G404–11. 10.1152/ajpgi.00088.2013. 24368703 10.1152/ajpgi.00088.2013 PMC3949020

[128] Carulli AJ, Keeley TM, Demitrack ES, Chung J, Maillard I, Samuelson LC. Notch receptor regulation of intestinal stem cell homeostasis and crypt regeneration. Dev Biol. 2015;402(1):98–108. 10.1016/j.ydbio.2015.03.012. 25835502 10.1016/j.ydbio.2015.03.012 PMC4433599

[129] Abud HE, Chan WH, Jardé T. Source and impact of the EGF family of ligands on intestinal stem cells. Front Cell Dev Biol. 2021;9:685665. 10.3389/fcell.2021.685665. 34350179 10.3389/fcell.2021.685665 PMC8327171

[130] Gao L, Zhong X, Jin J, Li J, Meng XM. Potential targeted therapy and diagnosis based on novel insight into growth factors, receptors, and downstream effectors in acute kidney injury and acute kidney injury-chronic kidney disease progression. Signal Transduct Target Ther. 2020;5(1):9. 10.1038/s41392-020-0106-1. 32296020 10.1038/s41392-020-0106-1 PMC7018831

[131] Zhou L, Zhou W, Joseph AM, Chu C, Putzel GG, Fang B, et al. Group 3 innate lymphoid cells produce the growth factor HB-EGF to protect the intestine from TNF-mediated inflammation. Nat Immunol. 2022;23(2):251–61. 10.1038/s41590-021-01110-0. 35102343 10.1038/s41590-021-01110-0 PMC8842850

[132] Zeng F, Harris RC. Epidermal growth factor, from gene organization to bedside. Semin Cell Dev Biol. 2014;28:2–11. 10.1016/j.semcdb.2014.01.011. 24513230 10.1016/j.semcdb.2014.01.011 PMC4037350

[133] Waterfield MD. Epidermal growth factor and related molecules. Lancet. 1989;1(8649):1243–6. 10.1016/s0140-6736(89)92339-8. 2566789 10.1016/s0140-6736(89)92339-8

[134] Holloway EM, Czerwinski M, Tsai YH, Wu JH, Wu A, Childs CJ, et al. Mapping development of the human intestinal niche at single-cell resolution. Cell Stem Cell. 2021;28(3):568-80.e4. 10.1016/j.stem.2020.11.008. 33278341 10.1016/j.stem.2020.11.008 PMC7935765

[135] Jardé T, Chan WH, Rossello FJ, Kaur Kahlon T, Theocharous M, Kurian Arackal T, et al. Mesenchymal niche-derived neuregulin-1 drives intestinal stem cell proliferation and regeneration of damaged epithelium. Cell Stem Cell. 2020;27(4):646-62.e7. 10.1016/j.stem.2020.06.021. 32693086 10.1016/j.stem.2020.06.021

[136] Basak O, Beumer J, Wiebrands K, Seno H, van Oudenaarden A, Clevers H. Induced quiescence of Lgr5+ stem cells in intestinal organoids enables differentiation of hormone-producing enteroendocrine cells. Cell Stem Cell. 2017;20(2):177-90.e4. 10.1016/j.stem.2016.11.001. 27939219 10.1016/j.stem.2016.11.001

[137] Zhang C, Jin Y, Marchetti M, Lewis MR, Hammouda OT, Edgar BA. EGFR signaling activates intestinal stem cells by promoting mitochondrial biogenesis and β-oxidation. Curr Biol. 2022;32(17):3704-19.e7. 10.1016/j.cub.2022.07.003. 35896119 10.1016/j.cub.2022.07.003 PMC10117080

[138] Wang J, Dempsey E, Corr SC, Kukula-Koch W, Sasse A, Sheridan H. The traditional Chinese medicine Houttuynia cordata Thunb decoction alters intestinal barrier function via an EGFR dependent MAPK (ERK1/2) signalling pathway. Phytomedicine. 2022;105:154353. 10.1016/j.phymed.2022.154353. 35932606 10.1016/j.phymed.2022.154353

[139] Wypych TP, Pattaroni C, Perdijk O, Yap C, Trompette A, Anderson D, et al. Microbial metabolism of L-tyrosine protects against allergic airway inflammation. Nat Immunol. 2021;22(3):279–86. 10.1038/s41590-020-00856-3. 33495652 10.1038/s41590-020-00856-3

[140] Snippert HJ, Schepers AG, van Es JH, Simons BD, Clevers H. Biased competition between Lgr5 intestinal stem cells driven by oncogenic mutation induces clonal expansion. EMBO Rep. 2014;15(1):62–9. 10.1002/embr.201337799. 24355609 10.1002/embr.201337799 PMC3983678

[141] Dahlhoff M, Horst D, Gerhard M, Kolligs FT, Wolf E, Schneider MR. Betacellulin stimulates growth of the mouse intestinal epithelium and increases adenoma multiplicity in Apc+/Min mice. FEBS Lett. 2008;582(19):2911–5. 10.1016/j.febslet.2008.07.026. 18656477 10.1016/j.febslet.2008.07.026

[142] Zhao H, Ren X, Kong R, Shi L, Li Z, Wang R, et al. Auxilin regulates intestinal stem cell proliferation through EGFR. Stem Cell Reports. 2022;17(5):1120–37. 10.1016/j.stemcr.2022.03.010. 35427486 10.1016/j.stemcr.2022.03.010 PMC9133653

[143] Hong AW, Meng Z, Guan KL. The Hippo pathway in intestinal regeneration and disease. Nat Rev Gastroenterol Hepatol. 2016;13(6):324–37. 10.1038/nrgastro.2016.59. 27147489 10.1038/nrgastro.2016.59 PMC5642988

[144] Fu M, Hu Y, Lan T, Guan KL, Luo T, Luo M. The Hippo signalling pathway and its implications in human health and diseases. Signal Transduct Target Ther. 2022;7(1):376. 10.1038/s41392-022-01191-9. 36347846 10.1038/s41392-022-01191-9 PMC9643504

[145] Moya IM, Halder G. Hippo-YAP/TAZ signalling in organ regeneration and regenerative medicine. Nat Rev Mol Cell Biol. 2019;20(4):211–26. 10.1038/s41580-018-0086-y. 30546055 10.1038/s41580-018-0086-y

[146] Chen L, Qin F, Deng X, Avruch J, Zhou D. Hippo pathway in intestinal homeostasis and tumorigenesis. Protein Cell. 2012;3(4):305–10. 10.1007/s13238-012-2913-9. 22492181 10.1007/s13238-012-2913-9 PMC4875478

[147] Gregorieff A, Wrana JL. Hippo signalling in intestinal regeneration and cancer. Curr Opin Cell Biol. 2017;48:17–25. 10.1016/j.ceb.2017.04.005. 28527754 10.1016/j.ceb.2017.04.005

[148] Deng H, Jia Q, Ming X, Sun Y, Lu Y, Liu L, et al. Hippo pathway in intestinal diseases: focusing on ferroptosis. Front Cell Dev Biol. 2023;11:1291686. 10.3389/fcell.2023.1291686. 38130953 10.3389/fcell.2023.1291686 PMC10734691

[149] Barry ER, Morikawa T, Butler BL, Shrestha K, de la Rosa R, Yan KS, et al. Restriction of intestinal stem cell expansion and the regenerative response by YAP. Nature. 2013;493(7430):106–10. 10.1038/nature11693. 23178811 10.1038/nature11693 PMC3536889

[150] Park HW, Kim YC, Yu B, Moroishi T, Mo JS, Plouffe SW, et al. Alternative Wnt signaling activates YAP/TAZ. Cell. 2015;162(4):780–94. 10.1016/j.cell.2015.07.013. 26276632 10.1016/j.cell.2015.07.013 PMC4538707

[151] Zhou D, Zhang Y, Wu H, Barry E, Yin Y, Lawrence E, et al. Mst1 and Mst2 protein kinases restrain intestinal stem cell proliferation and colonic tumorigenesis by inhibition of Yes-associated protein (Yap) overabundance. Proc Natl Acad Sci U S A. 2011;108(49):E1312–20. 10.1073/pnas.1110428108. 22042863 10.1073/pnas.1110428108 PMC3241824

[152] Gregorieff A, Liu Y, Inanlou MR, Khomchuk Y, Wrana JL. Yap-dependent reprogramming of Lgr5(+) stem cells drives intestinal regeneration and cancer. Nature. 2015;526(7575):715–8. 10.1038/nature15382. 26503053 10.1038/nature15382

[153] Jeng KS, Chang CF, Lin SS. Sonic Hedgehog signaling in organogenesis, tumors, and tumor microenvironments. Int J Mol Sci. 2020;21(3). 10.3390/ijms21030758. 10.3390/ijms21030758 PMC7037908 31979397

[154] Pontarollo G, Kollar B, Mann A, Khuu MP, Kiouptsi K, Bayer F, et al. Commensal bacteria weaken the intestinal barrier by suppressing epithelial neuropilin-1 and Hedgehog signaling. Nat Metab. 2023;5(7):1174–87. 10.1038/s42255-023-00828-5. 37414930 10.1038/s42255-023-00828-5 PMC10365997

[155] Walton KD, Gumucio DL. Hedgehog signaling in intestinal development and homeostasis. Annu Rev Physiol. 2021;83:359–80. 10.1146/annurev-physiol-031620-094324. 33035430 10.1146/annurev-physiol-031620-094324 PMC10278198

[156] Hanna J, Beke F, O’Brien LM, Kapeni C, Chen HC, Carbonaro V, et al. Cell-autonomous Hedgehog signaling controls Th17 polarization and pathogenicity. Nat Commun. 2022;13(1):4075. 10.1038/s41467-022-31722-5. 35835905 10.1038/s41467-022-31722-5 PMC9281293

[157] Frey MR. Sonic Hedgehog: powering up intestinal regeneration? Cell Mol Gastroenterol Hepatol. 2023;16(4):650–1. 10.1016/j.jcmgh.2023.07.010. 37562460 10.1016/j.jcmgh.2023.07.010 PMC10511917

[158] Kolterud A, Grosse AS, Zacharias WJ, Walton KD, Kretovich KE, Madison BB, et al. Paracrine Hedgehog signaling in stomach and intestine: new roles for hedgehog in gastrointestinal patterning. Gastroenterology. 2009;137(2):618–28. 10.1053/j.gastro.2009.05.002. 19445942 10.1053/j.gastro.2009.05.002 PMC2717174

[159] Orzechowska-Licari EJ, Bialkowska AB, Yang VW. Sonic Hedgehog and WNT signaling regulate a positive feedback loop between intestinal epithelial and stromal cells to promote epithelial regeneration. Cell Mol Gastroenterol Hepatol. 2023;16(4):607–42. 10.1016/j.jcmgh.2023.07.004. 37481204 10.1016/j.jcmgh.2023.07.004 PMC10470419

[160] Kosinski C, Stange DE, Xu C, Chan AS, Ho C, Yuen ST, et al. Indian hedgehog regulates intestinal stem cell fate through epithelial-mesenchymal interactions during development. Gastroenterology. 2010;139(3):893–903. 10.1053/j.gastro.2010.06.014. 20542037 10.1053/j.gastro.2010.06.014 PMC2930094

[161] Wang B, Shen J. NF-κB inducing kinase regulates intestinal immunity and homeostasis. Front Immunol. 2022;13:895636. 10.3389/fimmu.2022.895636. 35833111 10.3389/fimmu.2022.895636 PMC9271571

[162] Natarajan K, Abraham P, Kota R, Isaac B. NF-κB-iNOS-COX2-TNF α inflammatory signaling pathway plays an important role in methotrexate induced small intestinal injury in rats. Food Chem Toxicol. 2018;118:766–83. 10.1016/j.fct.2018.06.040. 29935243 10.1016/j.fct.2018.06.040

[163] Yang L, Li A, Wang Y, Zhang Y. Intratumoral microbiota: roles in cancer initiation, development and therapeutic efficacy. Signal Transduct Target Ther. 2023;8(1):35. 10.1038/s41392-022-01304-4. 36646684 10.1038/s41392-022-01304-4 PMC9842669

[164] Nabavi-Rad A, Sadeghi A, Asadzadeh Aghdaei H, Yadegar A, Smith SM, Zali MR. The double-edged sword of probiotic supplementation on gut microbiota structure in Helicobacter pylori management. Gut Microbes. 2022;14(1):2108655. 10.1080/19490976.2022.2108655. 35951774 10.1080/19490976.2022.2108655 PMC9373750

[165] Wang D, Odle J, Liu Y. Metabolic regulation of intestinal stem cell homeostasis. Trends Cell Biol. 2021;31(5):325–7. 10.1016/j.tcb.2021.02.001. 33648839 10.1016/j.tcb.2021.02.001

[166] Angelin A, Gil-de-Gómez L, Dahiya S, Jiao J, Guo L, Levine MH, et al. Foxp3 reprograms T cell metabolism to function in low-glucose. High-Lactate Environments Cell Metab. 2017;25(6):1282-93.e7. 10.1016/j.cmet.2016.12.018. 28416194 10.1016/j.cmet.2016.12.018 PMC5462872

[167] Bjarnason I, Scarpignato C, Holmgren E, Olszewski M, Rainsford KD, Lanas A. Mechanisms of damage to the gastrointestinal tract from nonsteroidal anti-inflammatory drugs. Gastroenterology. 2018;154(3):500–14. 10.1053/j.gastro.2017.10.049. 29221664 10.1053/j.gastro.2017.10.049

[168] Bensard CL, Wisidagama DR, Olson KA, Berg JA, Krah NM, Schell JC, et al. Regulation of tumor initiation by the mitochondrial pyruvate carrier. Cell Metab. 2020;31(2):284-300.e7. 10.1016/j.cmet.2019.11.002. 31813825 10.1016/j.cmet.2019.11.002 PMC7004878

[169] Khaloian S, Rath E, Hammoudi N, Gleisinger E, Blutke A, Giesbertz P, et al. Mitochondrial impairment drives intestinal stem cell transition into dysfunctional Paneth cells predicting Crohn’s disease recurrence. Gut. 2020;69(11):1939–51. 10.1136/gutjnl-2019-319514. 32111634 10.1136/gutjnl-2019-319514 PMC7569388

[170] Mihaylova MM, Cheng CW, Cao AQ, Tripathi S, Mana MD, Bauer-Rowe KE, et al. Fasting activates fatty acid oxidation to enhance intestinal stem cell function during homeostasis and aging. Cell Stem Cell. 2018;22(5):769-78.e4. 10.1016/j.stem.2018.04.001. 29727683 10.1016/j.stem.2018.04.001 PMC5940005

[171] Hou K, Wu ZX, Chen XY, Wang JQ, Zhang D, Xiao C, et al. Microbiota in health and diseases. Signal Transduct Target Ther. 2022;7(1):135. 10.1038/s41392-022-00974-4. 35461318 10.1038/s41392-022-00974-4 PMC9034083

[172] He J, Li H, Jia J, Liu Y, Zhang N, Wang R, et al. Mechanisms by which the intestinal microbiota affects gastrointestinal tumours and therapeutic effects. Mol Biomed. 2023;4(1):45. 10.1186/s43556-023-00157-9. 38032415 10.1186/s43556-023-00157-9 PMC10689341

[173] Claesson MJ, Jeffery IB, Conde S, Power SE, O’Connor EM, Cusack S, et al. Gut microbiota composition correlates with diet and health in the elderly. Nature. 2012;488(7410):178–84. 10.1038/nature11319. 22797518 10.1038/nature11319

[174] Lee YS, Kim TY, Kim Y, Lee SH, Kim S, Kang SW, et al. Microbiota-Derived Lactate Accelerates Intestinal Stem-Cell-Mediated Epithelial Development. Cell Host Microbe. 2018;24(6):833-46.e6. 10.1016/j.chom.2018.11.002. 30543778 10.1016/j.chom.2018.11.002

[175] Regnard C, Twycross R, Mihalyo M, Wilcock A. Loperamide. J Pain Symptom Manage. 2011;42(2):319–23. 10.1016/j.jpainsymman.2011.06.001. 21703817 10.1016/j.jpainsymman.2011.06.001

[176] Huang Y, Guo Y, Li X, Xiao Y, Wang Z, Song L, et al. Effects of Lactiplantibacillus plantarum GUANKE on diphenoxylate-induced slow transit constipation and gut microbiota in mice. Nutrients. 2023;15(17). 10.3390/nu15173741. 10.3390/nu15173741 PMC10490327 37686774

[177] Lamberts SWJ, Hofland LJ. ANNIVERSARY REVIEW: octreotide, 40 years later. Eur J Endocrinol. 2019;181(5):R173–83. 10.1530/eje-19-0074. 31398712 10.1530/EJE-19-0074

[178] Abtahi-Naeini B, Saffaei A, Sabzghabaee AM, Amiri R, Hosseini NS, Niknami E, et al. Topical sucralfate for treatment of mucocutaneous conditions: a systematic review on clinical evidences. Dermatol Ther. 2022;35(4):e15334. 10.1111/dth.15334. 35080090 10.1111/dth.15334

[179] Debono M, Price JN, Ross RJ. Novel strategies for hydrocortisone replacement. Best Pract Res Clin Endocrinol Metab. 2009;23(2):221–32. 10.1016/j.beem.2008.09.010. 19500765 10.1016/j.beem.2008.09.010

[180] Memar MY, Yekani M, Alizadeh N, Baghi HB. Hyperbaric oxygen therapy: antimicrobial mechanisms and clinical application for infections. Biomed Pharmacother. 2019;109:440–7. 10.1016/j.biopha.2018.10.142. 30399579 10.1016/j.biopha.2018.10.142

[181] Wenhui L, Changgeng F, Lei X, Baozhong Y, Guobin L, Weijing F. Hyperbaric oxygen therapy for chronic diabetic foot ulcers: an overview of systematic reviews. Diabetes Res Clin Pract. 2021;176:108862. 10.1016/j.diabres.2021.108862. 34015392 10.1016/j.diabres.2021.108862

[182] Sen S, Sen S. Therapeutic effects of hyperbaric oxygen: integrated review. Med Gas Res. 2021;11(1):30–3. 10.4103/2045-9912.310057. 33642335 10.4103/2045-9912.310057 PMC8103971

[183] Smit SG, Heyns CF. Management of radiation cystitis. Nat Rev Urol. 2010;7(4):206–14. 10.1038/nrurol.2010.23. 20212517 10.1038/nrurol.2010.23

[184] Leiper K, Morris AI. Treatment of radiation proctitis. Clin Oncol (R Coll Radiol). 2007;19(9):724–9. 10.1016/j.clon.2007.07.008. 17728120 10.1016/j.clon.2007.07.008

[185] Yang XF, Zheng MY, An LY, Sun JM, Hei QW, Ji YH, et al. Quality evaluation of guidelines for the diagnosis and treatment of radiation enteritis. Radiat Oncol. 2023;18(1):14. 10.1186/s13014-023-02204-9. 36670447 10.1186/s13014-023-02204-9 PMC9862547

[186] Lee JK, Agrawal D, Thosani N, Al-Haddad M, Buxbaum JL, Calderwood AH, et al. ASGE guideline on the role of endoscopy for bleeding from chronic radiation proctopathy. Gastrointest Endosc. 2019;90(2):171-82.e1. 10.1016/j.gie.2019.04.234. 31235260 10.1016/j.gie.2019.04.234

[187] Paquette IM, Vogel JD, Abbas MA, Feingold DL, Steele SR. The American society of colon and rectal surgeons clinical practice guidelines for the treatment of chronic radiation proctitis. Dis Colon Rectum. 2018;61(10):1135–40. 10.1097/dcr.0000000000001209. 30192320 10.1097/DCR.0000000000001209

